# Streetscape perception conditions the causal impacts of low-stress bicycle infrastructure

**DOI:** 10.1093/pnasnexus/pgag354

**Published:** 2026-11-10

**Authors:** M Mavin De Silva, Siqin Wang, Ashani Piyatilake, Sijian Tian, Santiago Garcia Gabilondo, Tadachika Nakayama

**Affiliations:** Extreme Energy-Density Research Institute, Nagaoka University of Technology, Nagaoka 940-2137, Japan; Department of Transport Management & Logistics Engineering, Faculty of Engineering, University of Moratuwa, Katubedda 10400, Sri Lanka; Center for Spatial Information Science, University of Tokyo, Kashiwa, Chiba, Japan; Spatial Sciences Institute, Dornsife College of Letters, Arts and Sciences, University of Southern California, Los Angeles, CA, USA; School of the Environment, University of Queensland, Brisbane, Australia; School of Science, RMIT University, Melbourne, VIC, Australia; Extreme Energy-Density Research Institute, Nagaoka University of Technology, Nagaoka 940-2137, Japan; Department of Civil Engineering, University of Tokyo, Hongo, Tokyo, Japan; Center for Spatial Information Science, University of Tokyo, Kashiwa, Chiba, Japan; Extreme Energy-Density Research Institute, Nagaoka University of Technology, Nagaoka 940-2137, Japan

**Keywords:** computational social science, causal inference, streetscape perception, bike lanes, safety

## Abstract

Perceived streetscapes (people’s visual evaluations of the surrounding street environment) influence how people respond to transportation infrastructure, yet their role in shaping cycling behavior remains underexplored. Here, we combine street-view imagery, spatial AI techniques, and causal inference to examine how neighborhood perceptions condition the effectiveness of bicycle infrastructure. We derive six types of perceptions from panoramic images and link them to built environment features. Leveraging the staggered rollout of protected and painted bike lanes in New York City, we implement a quasi-experimental, causal inferential framework using propensity score matching, difference-in-differences, and causal forest models on bikeshare origin–destination data. We find that bike lanes significantly increase ridership, with no detectable pretreatment effects. Protected lanes increase ridership by ∼32% (β=0.2705,p-value<0.001), while painted lanes increase ridership by ∼10% (β=0.0967,p-value<0.05). Treatment effects vary systematically across perceived environments: painted lanes perform best on already safe and lively streets, whereas protected lanes remain effective across a wider range of contexts and generate the strongest gains in stressful or lower-quality environments. Street-level counterfactual predictions provide the possibility to integrate human perception into infrastructure planning and design.

Significance Statement Understanding how bicycle infrastructure influences cycling activity is critical for sustainable mobility planning. Prior studies show that bike lanes can increase ridership, while a growing literature also demonstrates that cycling behavior is shaped by perceived safety, greenery, visual quality, and other human-scale streetscape characteristics. In this study, we propose a perception aware causal inference framework to evaluate not only whether bicycle lanes increase cycling, but also where and under what perceived neighborhood conditions they are most effective. Applied to bike lane installations in New York City, the framework uncovers substantial heterogeneity in bike-share trip responses across streetscapes. These findings provide practical guidance for designing bicycle networks that account for both infrastructure quality and human experience.

## Introduction

Increasing urban cycling is widely recognized as a key strategy to improve environmental sustainability, public health, and transportation safety. By reducing carbon emissions, air and noise pollution, and traffic-related fatalities, cycling offers substantial benefits compared to automobile-based travel (1–3). Despite these advantages, cycling counts in many cities remain low and unevenly distributed across populations, highlighting persistent barriers to adoption. Among the most effective interventions to promote cycling is the provision of bicycle infrastructure, particularly low-stress facilities such as protected bike lanes, which enhance both actual and perceived safety for riders (4). Therefore, a substantial body of research has focused on expanding and evaluating bike-lane networks, consistently finding that the presence and quality of infrastructure are key determinants of cycling uptake (5, 6).

Active-transportation research, both in the United States and internationally, provides consistent evidence of a positive association between bike lanes and bike ridership (2, 7). However, such research often overlooks a key component of cycling behavior (8, 9): the interaction between bicycle infrastructure and the perceived quality of surrounding streetscapes. The key question underlying this study is whether the effectiveness of bicycle infrastructure varies across different perceived streetscape conditions and facility types. In some contexts, favorable streetscape perceptions may reinforce infrastructure effects, while in others, stronger physical separation may provide greater benefits in less inviting environments. Understanding this heterogeneity is essential for designing cycling environments that are not only functional but also responsive to the aesthetic and psychological qualities of the surrounding streetscape (10, 11).

There are three important features of this existing literature that inform the design of this study. Prior work by Moran *et al.* (6) shows that protected bike lanes tend to generate larger increases in bikeshare ridership than painted lanes, and further argues that these benefits are not evenly distributed across space, with weaker effects observed in areas with higher proportions of non-White residents. These findings suggest that the impacts of bicycle infrastructure are inherently heterogeneous and shaped by underlying neighborhood characteristics. However, their identification strategy captures this heterogeneity primarily through coarse socio-demographic indicators, without accounting for how urban environments are experienced at the street level. In practice, responses to infrastructure are influenced not only by those who live in a neighborhood, but also by how that space is visually and perceptually interpreted (12). Ignoring these perceptual dimensions may lead to spatial imbalance between treated and control units, potentially biasing causal estimates and limiting the ability to explain observed heterogeneity fully. Second, while prior studies have leveraged street view imagery to quantify objective built environment characteristics and estimate their causal effects on cycling activity, they largely overlook how urban environments are perceived by individuals (5). This is a critical limitation, as behavioral responses to infrastructure are shaped not only by physical attributes but also by subjective experiences such as safety, livability, and visual appeal (13, 14). Third, existing causal analyses of cycling infrastructure typically focus on estimating average treatment effects, implicitly assuming that infrastructure interventions yield uniform impacts across space. Emerging observational evidence by Hwang *et al.* (8) suggests that the effectiveness of bicycle infrastructure may depend on environmental context, yet these relationships remain correlational and lack causal identification. To address the limitations identified in prior studies (5, 6, 8, 12), we propose a novel causal inference framework that leverages the staggered placement of bike lanes to examine how the effectiveness of bicycle infrastructure varies across perceptual contexts. This approach integrates deep learning-derived measures of perceived streetscapes with quasi-experimental methods, enabling robust identification of heterogeneous treatment effects and a deeper understanding of how infrastructure impacts vary across urban environments. Beyond its methodological contribution, the resulting street-level counterfactual predictions provide direct planning guidance, allowing practitioners to identify whether painted or protected lanes are likely to deliver the greatest ridership gains for a given street based on its perceptual conditions.

Similar to challenges identified in prior causal studies of built environment interventions (15, 16), the placement of bicycle infrastructure is often strategic and nonrandom, leading to potential biases arising from spatial imbalance between treated and control locations. To address this challenge while deepening our understanding of how bicycle infrastructure and human perception shape cycling behavior across diverse urban environments, this study leverages the staggered installation of low-stress (protected) and high-stress (painted) bike lanes across New York City (NYC), integrating large-scale bikeshare origin–destination (OD) data with street-level imagery to capture human-perceived streetscape qualities. Our analyses yield three key findings. First, both protected and painted bike lanes causally increase bikeshare ridership (6), with no evidence of pretreatment effects, supporting the validity of our identification strategy. Second, the magnitude of these effects varies systematically across perceptual environments: stronger increases are observed in neighborhoods perceived as safe, beautiful, and livable, while effects are attenuated in areas perceived as boring or depressing (17). Third, heterogeneous treatment effect estimation reveals that protected bike infrastructure provides more consistent benefits across a wider range of environments, while painted bike infrastructure is more sensitive to negative perceptual conditions (8). These findings are consistent with psychological theories of risk perception and environmental cognition, including prospect theory (18) and stress recovery theory (19, 20), which suggest that individuals’ travel behavior is shaped not only by physical safety but also by perceived comfort and stress. Together, our results demonstrate that the effectiveness of bicycle infrastructure depends critically on both its design and the perceived quality of the surrounding environment, offering new insights for human-centered transportation planning and smart city design.

## Results

The visual and functional characteristics of streets significantly impact how people perceive them and, consequently, how they use them. To capture this missing dimension of urban context, we assembled a large street-view imagery (SVI) dataset comprising 12,664 images collected from 3,166 sampling locations across NYC. Images were obtained through the Mapillary platform (21) and systematically sampled at 50-m intervals to reduce redundancy while preserving representative coverage across Manhattan, Brooklyn, Queens, and the Bronx. Figure 1a summarizes this spatial AI workflow used to quantify neighborhood perceptions from SVI. Using the MIT Place Pulse benchmark dataset (22), we estimated six dimensions of perceived streetscape quality relevant to travel behavior: beautiful, livable, safe, wealthy, boring, and depressing. Panoramic images were first decomposed into semantic visual elements using a PSPNet semantic segmentation model built on a ResNet-50 backbone (23), and these features were then linked to human perception scores using support vector machine models. Predictive performance was moderate to strong across all six dimensions (Figure 1b), indicating that machine learning can recover meaningful variation in how urban streets are visually experienced at city scale.

**Figure 1:**
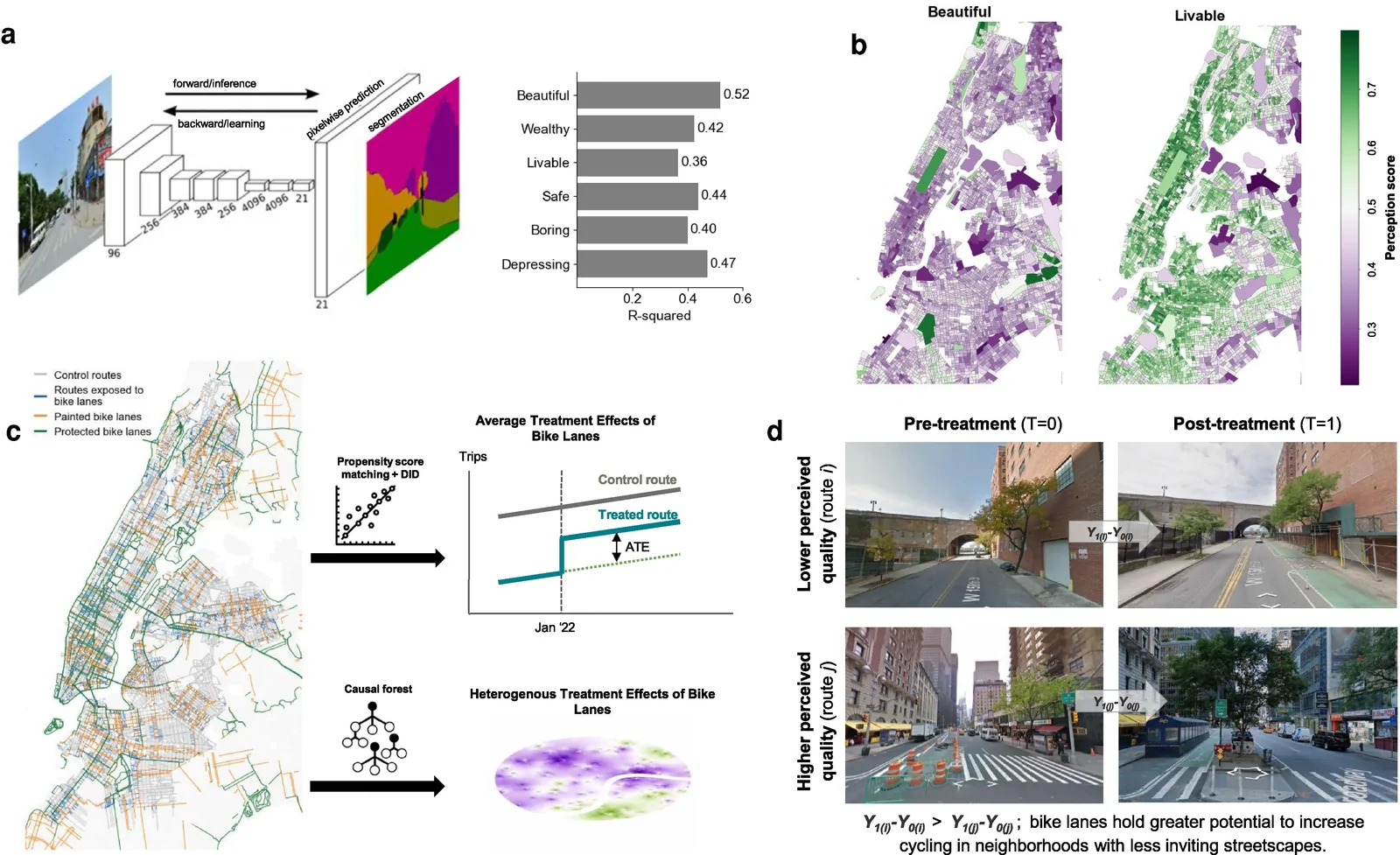
Panorama-derived neighborhood perceptions and causal study design. a) Extraction of human-perceived neighborhood attributes from street-level imagery. We employ a PSPNet semantic segmentation model with a ResNet-50 backbone to process panoramic images and derive pixel-level representations of the urban visual environment. These features are used to quantify six perception dimensions: beautiful, wealthy, livable, safe, boring, and depressing. A supervised learning framework (random forest regression) was employed to quantify the relationship between perceived neighborhood attributes and built environment features, with the strongest predictive performance observed for “beautiful” ($R^{2}=0.52$), followed by “depressing” ($R^{2}=0.47$) and “safe” ($R^{2}=0.44$). b) Spatial distribution of perceived neighborhood environments across NYC at the census block group level. The mapped indices reveal substantial spatial heterogeneity in perceived qualities, demonstrating that the model captures fine-grained, spatially coherent variation in urban perception. For clarity, only two dimensions (beautiful and livable) are shown here, while the remaining perception maps are provided in the online supplementary materials. c) Determinants of the causal inference framework, encompassing (1) spatial integration and (2) causal analysis. We first construct a detailed spatial representation of bike lane infrastructure, distinguishing protected lanes (physically separated), painted lanes (demarcated by paint), and sharrows (shared roadway markings). To address selection bias, we implement propensity score matching to pair bikeshare origin–destination routes that are similar in observed characteristics but differ in exposure to newly installed bike lanes. Leveraging the staggered timing of installations, we estimate average treatment effects using a difference-in-differences (DID) design, enabling causal identification under the parallel trends assumption. Finally, causal forest models are employed to estimate heterogeneous causal effects of bike lane interventions on cycling activity, enabling the identification of spatial and temporal variation beyond average treatment effects derived from the PSM–DID approach. d) Conceptual illustration of how streetscape perception moderates the impact of bicycle infrastructure. Four street-view images are shown: (1) low perceived quality streetscape before bike lane installation, (2) the same street after installation, (3) high perceived quality streetscape before installation, and (4) the same street after installation. Thus, images (1) and (3) represent baseline conditions, while (2) and (4) show post-treatment conditions. The comparison illustrates a compensatory pattern in which bicycle lanes, particularly low-stress facilities, tend to generate larger ridership gains in less inviting environments, suggesting that infrastructure can help offset adverse street conditions and promote more equitable cycling access. Street-view images were obtained from Mapillary and are reproduced in accordance with the applicable Mapillary terms of use. Maps were produced in Python using TIGER shapefiles from the US Census Bureau.

We then integrated these perception measures into a causal framework to test whether the same bicycle infrastructure performs differently across neighborhood contexts. OD Citi Bike routes were classified according to whether they intersected newly installed painted or protected bike lanes, while untreated routes served as candidate controls (see Methods and online supplementary material Section 2.1 for more details on linking Citi Bike trip data with bike lane data). Routes were matched on pretreatment ridership and comparable pretreatment socio-economic, network, and perceptual characteristics, followed by staggered difference-in-differences (DID) and causal forest estimation (Figure 1c). The conceptual framework in Figure 1d illustrates the central hypothesis that streetscape quality moderates infrastructure effectiveness. Our results support a compensatory pattern in which bicycle lanes, particularly low-stress facilities, tend to generate larger ridership gains in less inviting environments, suggesting that infrastructure can offset adverse street conditions and help expand cycling more equitably across neighborhoods.

Before estimating treatment effects, we first assess whether treated and control bikeshare routes are comparable in their pretreatment characteristics. Unlike conventional matching approaches (4, 6) that focus on a single station or corridor, our unit of analysis is the OD trip, requiring balance at both origins and destinations. This distinction matters because cycling demand depends jointly on where trips begin and end. Failing to account for this bidirectional structure may introduce selection bias, as routes connected to central, affluent, or already bike-friendly destinations are more likely to receive infrastructure and exhibit higher ridership independent of treatment.

To address this challenge, we develop a staggered route-matching framework combining phased bike-lane installation with rich OD-level covariates. Using propensity score matching (PSM), treated routes are paired with untreated routes exhibiting similar pretreatment ridership and comparable origin and destination characteristics, including population density, road and intersection density, walkability, racial composition, household income, unemployment, activity density, and machine learning-derived perception measures such as safety, beauty, wealth, livability, boringness, and depressingness. Figure 2a shows that treated and untreated routes display limited propensity-score overlap before matching, consistent with strategic bike-lane placement along already favorable corridors. Figure 2b further shows that origin- and destination-level covariate differences converge toward zero (−0.1<covariate difference<0.1), indicating that our staggered OD matching strategy successfully constructs a credible counterfactual set of comparison routes, substantially reducing observable selection bias before implementing the DID analysis (see Methods and online supplementary material Section 2.2 for more details on the PSM approach). The OD-pair representation also preserves destination-specific variation in infrastructure exposure that would be obscured by origin-station aggregation. In an additional route-network diagnostic, 69.4% of origin stations represented by at least two sampled OD pairs were connected to routes spanning two or more dominant bicycle-infrastructure classes. The median within-origin range in overall bicycle-infrastructure exposure was 0.480, while the corresponding range in protected-lane exposure was 0.336. Thus, routes departing from the same Citi Bike station can encounter substantially different infrastructure environments depending on their destinations, providing a substantive rationale for retaining the OD-pair unit. We further assessed sensitivity to the single-shortest-path assumption using a stochastic multi-route representation. Although 98.3% of successfully routed OD pairs had multiple plausible routes, protected-lane exposure remained highly consistent between the deterministic and stochastic representations (Spearman ρ=0.969, MAE=0.028), as did overall bicycle-infrastructure exposure (ρ=0.964, MAE=0.048); the dominant infrastructure class agreed for 93.1% of OD pairs (online supplementary Note 2.4). Although matching improves balance on observed characteristics, it cannot account for unobserved time-varying confounders, and the inferred OD routes represent plausible exposure paths rather than observed cyclist trajectories. The resulting estimates should therefore be interpreted within these identification and exposure-measurement assumptions.

**Figure 2:**
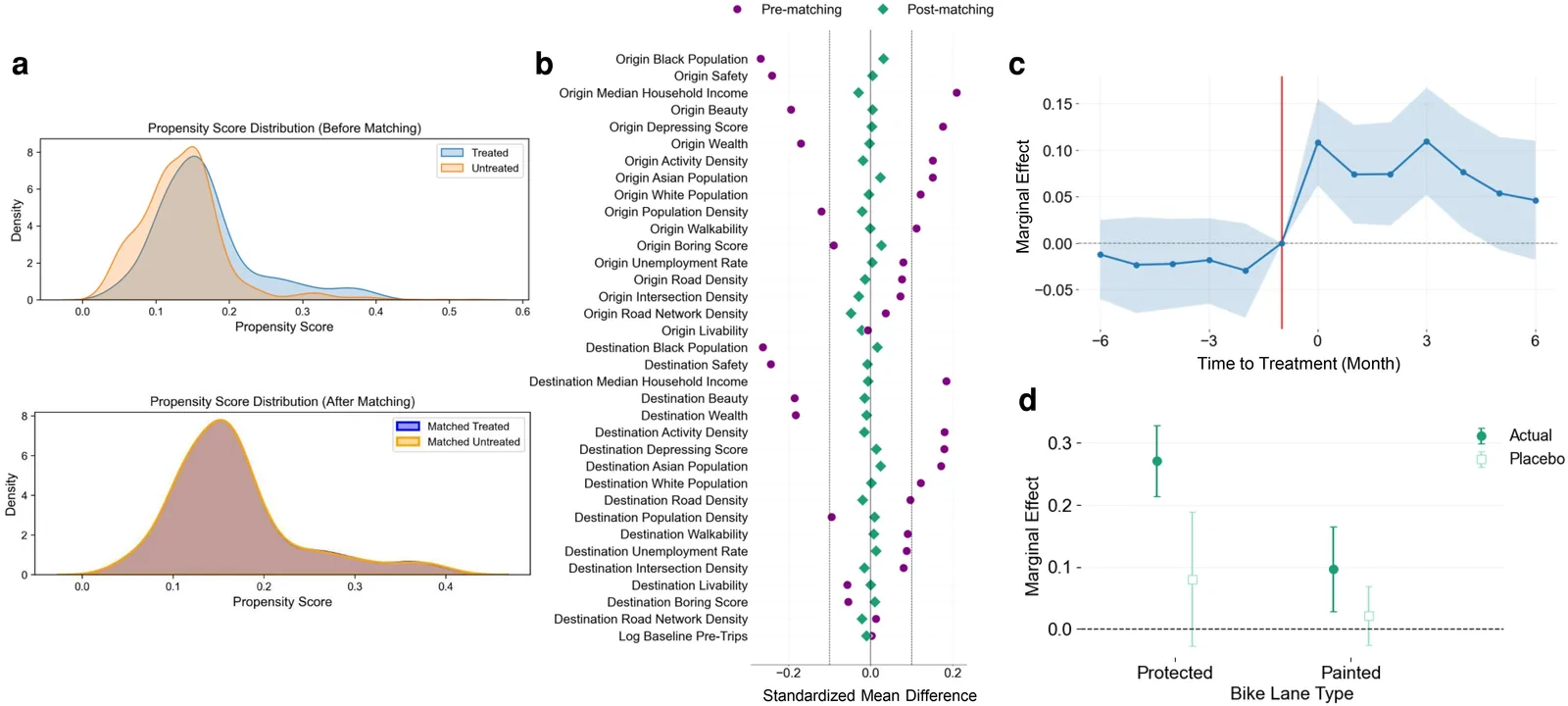
Causal estimation of bicycle lane impacts across facility types. a) Distribution of propensity scores for treated and control routes before and after matching. Before matching, the two groups show limited overlap, reflecting the nonrandom placement of bike lanes along corridors already favorable to cycling. After matching, the distributions align closely, indicating improved common support and enhanced comparability between treated and control routes. b) Graphical representation of origin- and destination-level covariate balances before and after propensity score matching. c) Time variation of the treatment effects on average ridership among matched bikeshare OD pairs analyzed through event study in NYC, with the center of shaded error bands representing point estimates and error bands representing the 95% confidence intervals. The red vertical line represents the baseline period, which is one month before the treatment. Effects were statistically insignificant prior to bike lane installation, supporting the parallel trend assumption of the DID strategy, but turned significantly positive afterward, highlighting bike lane installations increase average ridership. d) DID analysis of the change in bikeshare trips following the installation of protected bike lanes (left) and painted bike lanes and sharrows (right). The significant DID estimates highlight its robustness in capturing the causal impacts of both protected and painted bike lanes on ridership.

### Placement of bike lanes causally increases cycling activity, with stronger gains from low-stress facilities

We begin our analysis by evaluating the causal impact of bicycle lane installation on bikeshare activity using a staggered quasi-experimental design. To examine the temporal evolution of these effects, we first implement an event study framework (described in the Methods section), exploiting the phased rollout of bicycle infrastructure across NYC. Figure 2c shows the estimated treatment effects on average ridership for matched OD routes before and after exposure to newly installed bike lanes. We observe no statistically significant pretreatment effects, supporting the parallel-trends assumption. Following installation, ridership rises significantly by ∼12% (p-value<0.001), over time, indicating structural shifts in travel behavior rather than short-term novelty responses. These findings are consistent with theories of induced demand and latent cycling potential, whereby safer and more connected infrastructure lowers barriers to bicycle adoption (9, 24, 25). Given the distinction between facility designs, we next estimate average treatment effects separately for protected and painted lanes using: $\text{ln}(Y_{it})=\alpha +\beta_{0}W_{it}.1(C_{i}=0)+\beta_{1}W_{it}.1(C_{i}=1)+\mu_{i}+\lambda_{t}+\varepsilon_{it}$. where $Y_{it}$ is “number of bike trips + 1” observed on route *i* in month *t*. $W_{it}$ is a binary indicator equal to 1 if route *i* is exposed to bicycle infrastructure and the corresponding installation year and month have been reached, and 0 otherwise. The variable $C_{i}\in \{0,1\}$ denotes the type of bike lane treatment assigned to route *i*, where $C_{i}=0$ indicates a painted bike lane and $C_{i}=1$ indicates a protected bike lane. The coefficient $\beta_{0}$ therefore captures the average treatment effect of painted bike lanes on logged ridership, while $\beta_{1}$ captures the effect of protected bike lanes. $\mu_{i}$ accounts for route-level fixed effects, controlling for time-invariant differences across OD routes, and $\lambda_{t}$ represents time fixed effects (year and month), capturing common temporal shocks affecting all routes. Finally, $\varepsilon_{it}$ denotes the error term.

Figure 2d summarizes the DID estimates by lane type. Protected bike lanes generate the largest gains, increasing ridership by ∼32% (β=0.2705,p-value<0.001), whereas painted lanes increase ridership by ∼10% (β=0.0967,p-value<0.05). These findings indicate that physical separation from traffic provides benefits beyond lane demarcation alone, reinforcing that infrastructure quality strongly shapes cycling outcomes. The full regression tables are provided in Table S5. To test whether these effects could arise from spurious trends, we conduct placebo tests by randomly shuffling installation dates while holding route characteristics fixed. Across 10 placebo datasets, estimated effects are statistically insignificant and centered near zero (Figure 2d), supporting the interpretation that the positive ridership gains identified in the main analysis are attributable to actual bike-lane installation rather than coincident shocks or model artifacts (see Methods and online supplementary material).

### Perceived streetscape quality shapes heterogeneous cycling responses to infrastructure

We next examine whether perceived streetscape quality moderates infrastructure effectiveness using a multi-arm causal forest model. Whereas staggered DID identifies average treatment effects, it does not reveal whether responses vary across neighborhoods. In practice, the same bike lane may generate large gains in one corridor but limited gains in another depending on built form, socio-economic context, and perceived safety or attractiveness. To uncover this variation, we implement a causal random forest estimator that jointly models treatment assignment and outcome dynamics while allowing flexible nonlinear interactions among route characteristics. Prior studies combine PSM and causal forests to study heterogeneous links between the built environment and cycling counts (5, 26), but typically rely on contemporaneous outcomes rather than treatment-timing shocks. By contrast, our framework applies a multi-arm causal forest to staggered matched route pairs using pre–post ridership changes around actual infrastructure deployment events.

Using matched treated and control route pairs, we define the outcome variable as the change in logged ridership between the post-treatment and pretreatment periods, $\Delta \text{ln}(Y_{i})=\text{ln}({\text{PostTrips}}_{i})-\text{ln}({\text{PreTrips}}_{i})$. We then relate this outcome to treatment status and route characteristics within the causal forest framework. The treatment assignment variable, $W_{i}$, identifies the infrastructure condition associated with route *i* and is specified as a multi-valued treatment indicator: $W_{i}\in \{0,1,2\}$, where $W_{i}=0$ denotes matched control routes with no new bike-lane exposure, $W_{i}=1$ denotes routes exposed to painted bike lanes, and $W_{i}=2$ denotes routes exposed to protected bike lanes. The causal forest then estimates the conditional average treatment effect (CATE), $\tau_{k}(x)=E[Y_{i}(k)-Y_{i}(0)|X_{i}=x],\;k\in \{1,2\},$ which measures how the effect of bicycle infrastructure varies across routes with different contextual characteristics. Here, $Y_{i}(k)$ denotes the potential outcome for route *i* under treatment type *k* (painted or protected lanes), while $Y_{i}(0)$ represents the potential outcome under no new infrastructure. The vector $X_{i}$ includes baseline ridership plus origin- and destination-level contextual variables (see Methods and online supplementary material Section 3.1 for more details on the CATE). This framework captures how infrastructure performance depends on both lane type and neighborhood conditions. The causal forest estimates closely mirror the matched DID results, providing an important validation of the main causal findings. Protected lanes increase ridership by approximately ∼32 % (β=0.2763,p-value<0.05), whereas painted lanes increase ridership by ∼8%  (β=0.0771,p-value<0.05). The convergence of these estimates across two distinct methodological frameworks, one based on quasi-experimental panel comparisons and the other on nonparametric heterogeneous effect learning, suggests that the benefits of bicycle infrastructure are robust rather than model-specific. They also indicate a robust and persistent empirical pattern in which lower-stress facilities deliver substantially greater cycling gains than higher-stress painted bike lanes.

To clarify which perceptual dimensions explain this heterogeneity, we estimate separate moderation models by lane type l∈{painted,protected}: $\tau_{i}^{\left(l\right)}=\alpha^{\left(l\right)}+\sum_{k=1}^{6}\beta_{k}^{\left(l\right)}z_{ik}+\varepsilon_{i}$, where $\tau_{i}^{\left(l\right)}$ denotes the estimated treatment effect for route *i* under lane type *l*, and $z_{ik}$ represents the standardized score of each perception dimension *k*. Figure 3a reports the resulting moderation coefficients. First, perceived livability positively moderates both lane types, with larger effects for protected lanes (β=0.255,p<0.001) than painted lanes (β=0.200,p<0.001). Second, perceptions of beauty strengthen infrastructure effectiveness primarily for protected lanes (β=0.157,p<0.001), while the painted-lane effect is small and insignificant (β=0.027,p=0.128). Third, perceived safety exhibits contrasting relationships across lane types. For painted lanes, higher perceived safety is associated with larger treatment effects (β=0.067,p<0.001), whereas for protected lanes the relationship is negative (β=−0.098,p<0.001). By contrast, perceived boringness is one of the strongest positive moderators for both painted (β=0.234,p<0.001) and protected lanes (β=0.276,p<0.001). This result suggests that bicycle infrastructure can generate especially large gains in visually monotonous or low stimulation environments, potentially by improving mobility opportunities where existing street conditions are otherwise less inviting. Wealth shows little effect, while depressingness is positive only for painted lanes (β=0.081,p=0.002). Taken together, these findings indicate that infrastructure benefits depend not only on engineering design but also on how neighborhoods are psychologically experienced. The full regression tables are provided in Table S9.

**Figure 3:**
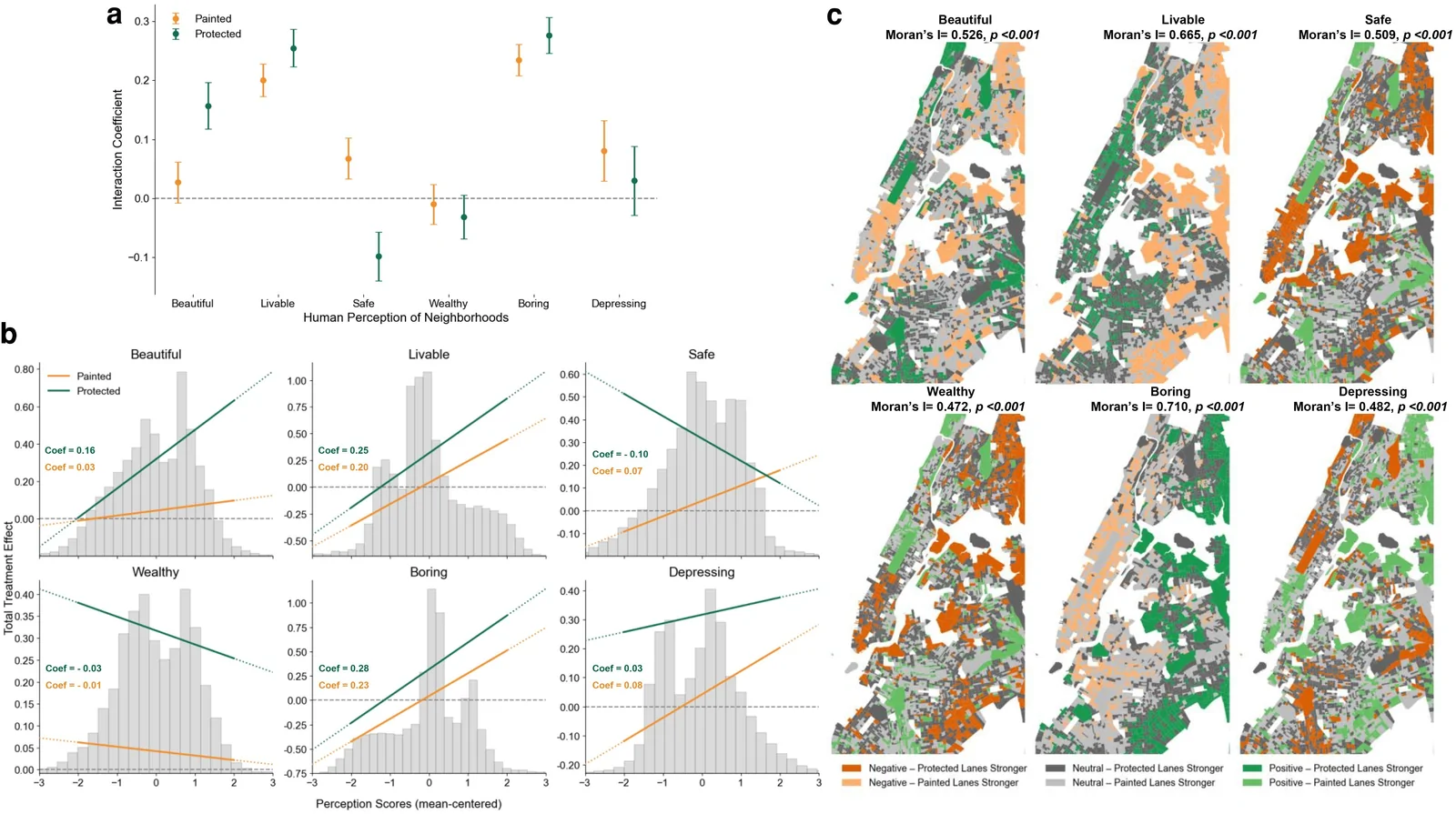
Interaction effects of street perceptions on bicycle lane impacts. a) This plot with error bars illustrates how the effectiveness of bicycle lanes varies across different types of neighborhood environments. The *x*-axis represents six neighborhood perception categories including beautiful, livable, safe, wealthy, boring, and depressing. The *y*-axis displays the estimated contribution of each perception to the overall bike lane effect, holding all other perceptions constant. In practical terms, these values indicate how much more (or less) effective bicycle lanes are in neighborhoods characterized by higher levels of a given perception relative to others. Positive coefficients indicate that bicycle lanes tend to be more effective in neighborhoods where that perception is more prominent, while negative values indicate reduced effectiveness. Error bars represent 95% confidence intervals around the estimated effect of each perception on bike lane effectiveness. b) We translate estimated marginal effect coefficients into predicted treatment effects across the observed distribution of perception scores, revealing how bike lane effectiveness varies across neighborhood contexts. Each panel shows one perception dimension, with the *x*-axis represents standardized (mean-centered) perception scores, where $z=\frac{x-\mu }{\sigma }$, with zero denoting the citywide average and positive (negative) values indicating neighborhoods perceived more (less) strongly along that dimension. The *y*-axis shows the predicted bike lane effect, representing the estimated causal impact of bike lane installation conditional on perception levels. Lines denote painted (orange) and protected (green) lanes, illustrating how their impacts vary across perception levels, while background histograms indicate the distribution of neighborhoods along each perception dimension, providing context for where observations are concentrated. c) We map the spatial distribution of perception conditioned treatment effects across NYC census block groups. Each area is classified by its perceptual context (negative, neutral, or positive) and the lane type associated with a stronger predicted effect, synthesizing the continuous relationships shown in panel (b) into discrete spatial patterns. All six perception dimensions exhibit significant positive spatial autocorrelation (Moran’s I=0.472 to 0.710, permutation p<0.001), indicating that neighboring CBGs tend to exhibit similar perception-conditioned relative advantages of protected versus painted lanes rather than random spatial dispersion. Maps were produced in Python using the TIGER shapefiles from the US Census Bureau.

To translate these coefficients into interpretable treatment effects, we next compute predicted treatment gains as ${\hat{\tau }}^{\left(l\right)}(z_{k})={\hat{\alpha }}^{\left(l\right)}+{\hat{\beta }}_{k}^{\left(l\right)}z_{k},$ where $z_{k}$ denotes the standardized perception score, such that $z_{k}=0$ corresponds to the citywide average, positive values indicate neighborhoods perceived more strongly along that dimension, and negative values indicate neighborhoods perceived less strongly. Baseline effects are substantially larger for protected lanes $\left(\hat{\alpha }=0.317,p<0.001\right)$ than painted lanes $\left(\hat{\alpha }=0.042,p<0.001\right)$, indicating that even before accounting for perceptual context, low-stress facilities generate markedly stronger cycling gains. Figure 3b extends this comparison by tracing predicted treatment effects for both lane types across the observed distribution of neighborhood perception scores, with background histograms indicating where perceptions are most commonly concentrated. The protected lanes outperform painted lanes across most perceptual contexts, though the magnitude varies systematically with neighborhood experience.

For perceived beauty, treatment effects rise steeply for protected lanes (β=0.157,p<0.001), while the effect for painted lanes is small and statistically insignificant (β=0.027,p=0.128), indicating that visually attractive environments primarily enhance the returns to physically separated infrastructure (27–29). A similar pattern is observed for livability, with positive effects for both lane types but larger for protected lanes (β=0.255) than painted lanes (β=0.200), both p<0.001, suggesting the greatest gains occur in highly livable neighborhoods. Perceived safety shows the strongest divergence: painted-lane effects increase with higher safety (β=0.067,p<0.001), whereas protected-lane effects decrease (β=−0.098,p<0.001), indicating that the effectiveness of protected lanes diminishes in already safe environments and is greatest in neighborhoods perceived as less safe. Perceived wealth has weak and insignificant effects for both lane types. By contrast, boringness strongly increases treatment effects for both painted (β=0.234,p<0.001) and protected lanes (β=0.276,p<0.001), suggesting that infrastructure is particularly effective in visually monotonous settings (28, 30). For depressing environments, both lane types show positive predicted gradients, stronger for painted lanes (β=0.081,p=0.002), while the relative advantage for protected-lane effect is smaller and insignificant.

### Cycling responses are geographically clustered and visible at the street level

Having shown that perceived streetscape quality moderates infrastructure effectiveness, we next examine how these heterogeneous responses are distributed spatially. While Figure 3a and b summarize aggregate relationships, Figure 3c maps them across NYC Census Block Groups (CBGs), revealing where neighborhood context amplifies the relative performance of protected versus painted lanes. For each perception dimension *k*, we compute the perception-conditioned relative advantage of protected over painted lanes as $\Delta_{k}(z_{k})={\hat{\tau }}_{k}^{\left(R\right)}(z_{k})-{\hat{\tau }}_{k}^{\left(P\right)}(z_{k})=({\hat{\beta }}_{k}^{\left(R\right)}-{\hat{\beta }}_{k}^{\left(P\right)})z_{k},$ where $z_{k}$ denotes the standardized perception score for dimension *k*, and superscripts *R* and *P* refer to protected and painted lanes, respectively. Positive values of $\Delta_{k}(z_{k})$ indicate a relative advantage for protected lanes, while negative values favor painted lanes. Because intercepts are excluded, this measure captures differential sensitivity rather than absolute treatment effects. To aid interpretation, each census block group is further classified according to whether the standardized perception score is negative $\left(z_{k}<-1\right)$, neutral $\left(-1\leq z_{k}\leq 1\right)$, or positive $\left(z_{k}>1\right)$, yielding six spatial categories that jointly summarize lane advantage and contextual intensity.

The six perception dimensions exhibit distinct spatial patterns across NYC and should not be interpreted as direct proxies for one another. For example, streets perceived as beautiful are not necessarily perceived as livable or wealthy, while low boringness does not necessarily imply greater livability. Although greenery, buildings, roads, and other visible streetscape characteristics may contribute to multiple dimensions, their relationships with individual perceptions are not equivalent (31). Accordingly, the relatively low boringness observed across much of Manhattan is interpreted as a pattern of visual perception rather than attributed to a single physical characteristic. These measures are therefore used primarily to characterize the broader visual context surrounding bicycle infrastructure.

Formal spatial-autocorrelation analysis further confirms that the model-implied CBG-level patterns in Figure 3c are nonrandomly spatially structured. Global Moran’s *I* ranges from 0.472 to 0.710 across the six dimensions (all permutation-based p<0.001), while local Moran’s *I* identifies significant high–high and low–low clusters (online supplementary Note 3.3). For beauty and livability, relative advantages of protected lanes are concentrated in Manhattan and western Brooklyn. For perceived safety, protected lanes are more prominent in negatively perceived locations, particularly along busy corridors (32, 33), while painted lanes become more competitive in already safe environments (34, 35). For boringness and depressingness, areas favoring protected lanes appear in eastern Manhattan, the South Bronx, and parts of outer Brooklyn and Queens. Nonetheless, some neighborhoods favor painted lanes, highlighting considerable spatial variation in the relative performance of the two infrastructure types.

Building on the CBG-level spatial patterns shown in Figure 3c, Figure 4 translates the estimated perception-conditioned treatment relationships to selected street-level scenarios. Whereas Figure 3c uses CBG-level perception scores to illustrate how the relative advantage of protected versus painted lanes varies spatially across NYC, Figure 4 applies the fitted multivariate moderation coefficients to the local perception profile of each selected street segment. For each segment *s*, the six perception scores are standardized using the same citywide scaling parameters used in model estimation and entered into the fitted treatment-effect function, ${\hat{\tau }}_{s}^{\left(l\right)}={\hat{\alpha }}^{\left(l\right)}+\sum_{k=1}^{6}{\hat{\beta }}_{k}^{\left(l\right)}z_{sk}$ where ${\hat{\alpha }}^{\left(l\right)}$ is the baseline effect for lane type *l*, ${\hat{\beta }}_{k}^{\left(l\right)}$ is the estimated moderation coefficient for perception dimension *k*, and $z_{sk}$ is the standardized perception score of segment *s*. Thus, the street-level values in Figure 4 are calculated directly from the fitted moderation model and segment-specific perception profiles, rather than being transferred from the CBG-level values in Figure 3c. Figure 4b shows the visual context of the selected corridors, while Figure 4c reports the corresponding model-implied ridership gains under hypothetical painted- and protected-lane scenarios.

**Figure 4:**
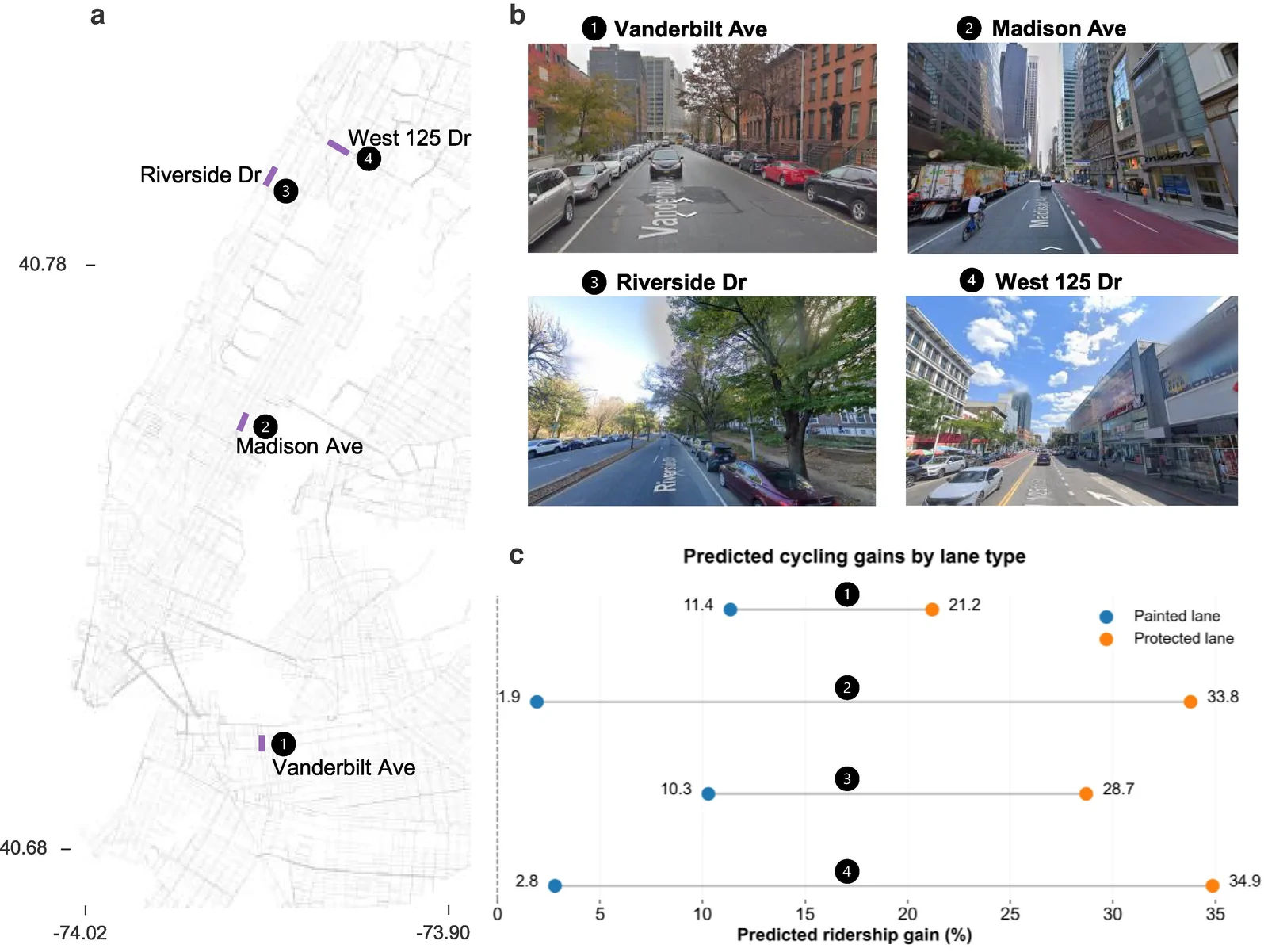
Street-level scenario analysis of bicycle lane design across selected urban corridors. a) Locations of four candidate street segments in NYC used for counterfactual analysis. Numbered labels correspond to (1) Vanderbilt Avenue, (2) Madison Avenue, (3) Riverside Drive, and (4) West 125th Street. b) Street view images illustrating local streetscape conditions for the selected corridors. Images highlight variation in urban form, traffic intensity, and perceived environment across locations, providing visual context for the predicted treatment effects. c) Model-implied ridership gains under hypothetical painted- and protected-lane scenarios for each selected corridor. Predictions are generated by applying the fitted OD-route-level moderation model to the standardized perception profile of each street segment, illustrating how estimated perception-conditioned relationships can be translated into street-level planning scenarios.

For the street-level scenario analysis, the four corridors represent contrasting combinations of visually perceived safety and overall neighborhood soundness. Vanderbilt Avenue and Riverside Drive represent relatively higher-safety environments, while differing in their broader neighborhood-soundness profiles. Madison Avenue and West 125th Street represent relatively lower-safety environments, again spanning higher and lower neighborhood soundness. These four combinations provide a simple typology for comparing how painted- and protected-lane scenarios perform across different perceived street environments (online supplementary Note 3.5). Across the four corridors, the effectiveness of painted versus protected lanes varies substantially with local streetscape conditions, highlighting that infrastructure performance is not spatially uniform. Vanderbilt Avenue and Riverside Drive, both relatively lower-stress corridors, show more modest gaps between lane types. Vanderbilt Avenue is associated with gains of 11.4% under painted treatment and 21.2% under protected treatment, while Riverside Drive shows 10.3% and 28.7%, respectively. Although protected lanes still outperform, the modest differences suggest that in already low-stress or more comfortable environments, painted lanes can still deliver meaningful ridership gains. On the flip side, Madison Avenue in Midtown Manhattan and West 125th Street both exhibit large divergences between lane types, with painted treatments yielding only 1.9% and 2.8% increases, compared with 33.8% and 34.9% under protected treatment, respectively. Within the fitted model, these scenarios illustrate how perception-conditioned treatment relationships translate into different expected ridership responses across selected street contexts, providing a policy-translation framework for comparing painted and protected lane alternatives.

## Discussion

Estimating the causal effect of bicycle infrastructure on urban travel behavior presents a complex challenge, particularly in dense cities where lane placement is rarely random. New bike lanes are often installed along corridors with rising demand, favorable land uses, or pre-existing cycling activity, creating endogeneity concerns that complicate causal inference. This makes it difficult to determine whether ridership growth is driven by infrastructure itself or by underlying street characteristics. Resolving this issue is essential not only to justify public investment, but also to guide equitable and evidence-based mobility planning as cities pursue decarbonization and congestion reduction goals. The challenge is especially pronounced in large metropolitan areas, where competition for roadway space is intense and returns to infrastructure vary across neighborhoods. More broadly, there remains a need for causal frameworks that account for both the route-based nature of cycling demand and the lived experience of streets.

In this context, this study makes four key contributions. First, we introduce a staggered matching framework that accounts for the timing and location of bike-lane deployment while representing infrastructure exposure at the OD-route level. This representation complements station-centered approaches by preserving destination-specific variation in the infrastructure encountered between trip origins and destinations. Our route-network diagnostic confirms that this distinction is substantively meaningful: 69.4% of origin stations serving multiple sampled destinations were associated with routes spanning more than one dominant infrastructure class, with a median within-origin range of 0.336 in protected-lane exposure. Staggered matching, in turn, improves comparability between treated and control routes on observed pretreatment characteristics. By leveraging phased rollout dates and matching routes on pretreatment ridership and OD characteristics, the framework reduces bias from spatial sorting and network expansion. This is particularly important in dense cities like New York, where infrastructure is often installed in already favorable corridors. Second, we show that infrastructure quality matters substantially for cycling uptake. The staggered Difference-in-Differences model estimates that protected lanes increase ridership by approximately 32%  (β=0.2705), whereas painted lanes increase ridership by roughly 10%  (β=0.0967). These results are closely mirrored by the causal forest estimates, which imply average gains of 32% for protected lanes and 8.0% for painted lanes. The consistency of these findings across quasi-experimental and machine-learning frameworks suggests that the superior performance of protected infrastructure is not an artifact of model choice, but a robust empirical pattern (32, 33, 36).

Third, we demonstrate that infrastructure effectiveness varies systematically with perceived streetscape conditions. Using machine learning-derived measures of safety, beauty, livability, wealth, boringness, and depressingness, we show that treatment effects are highly context dependent. The baseline treatment effect, measured at average citywide perception levels, is substantially larger for protected lanes $\left(\hat{\alpha }=0.317,p<0.001\right)$ than for painted lanes $\left(\hat{\alpha }=0.042,p<0.001\right)$, indicating that even in a typical streetscape environment, low-stress facilities generate markedly stronger gains. Relative changes suggest that painted lanes perform best where streets already feel safe and active, whereas protected lanes remain effective in more stressful or visually unattractive settings (32, 33, 36), suggesting that physical separation can compensate for adverse conditions. These findings highlight the importance of context-sensitive planning rather than one-size-fits-all approaches. Fourth, we translate these estimates into street-level planning scenarios. Predicted gains vary considerably across corridors, highlighting the importance of local context. Vanderbilt Avenue and Riverside Drive, both relatively lower-stress environments, show more modest differences between treatments, indicating that painted lanes can still deliver meaningful gains where baseline conditions shaped by visual cues such as graffiti, lighting, and urban design are already supportive (34, 35, 37). In contrast, Madison Avenue and West 125th Street exhibit much larger gaps, with protected lanes substantially outperforming painted alternatives. These high-traffic, high-demand corridors underscore the importance of physical separation in reducing stress and unlocking cycling demand (32, 36). Together, these examples demonstrate how causal estimates can guide context-sensitive infrastructure planning, aligning lane design with local street conditions.

This context-sensitive interpretation is consistent with evidence from mature cycling cities and bicycle facility design guidance internationally. In cities such as Amsterdam and Copenhagen, high cycling participation is supported by networks that combine separated cycling facilities on major corridors (32) with calmer local streets where low speeds, low traffic volumes, and high perceived comfort reduce the need for heavy physical separation. NACTO’s bikeway design guidance similarly emphasizes that not every segment of a bicycle network requires a protected lane; where vehicle speeds and volumes are low and cycling stress is already limited, less intensive treatments can still support comfortable cycling (38). Our results extend this logic by showing that perceived streetscape quality provides an additional layer for facility selection: painted lanes can remain useful where streets already exhibit high perceived safety, calmer traffic conditions, and visually supportive environments, whereas protected lanes are more critical where traffic stress or perceived risk suppresses cycling demand. Thus, the planning implication is not that one lane type should be applied uniformly, but that facility choice should be conditioned on both traffic stress and perceived streetscape quality.

While our staggered OD matching framework improves comparability on observed characteristics and leverages variation in the timing of bike-lane deployment, it does not eliminate potential bias from unobserved time-varying factors that may influence both infrastructure placement and ridership trends (39). Future studies could complement this design with alternative identification strategies, such as instrumental-variable or synthetic-control approaches, where appropriate. Second, our route exposure measure is based on the optimal time-distance bicycle path between each OD pair as generated through OSMnx and NetworkX, which approximate travel behavior but may not fully capture actual route choice. Integrating GPS or app-based trajectory data would improve measurement of experienced infrastructure exposure. Third, bicycle infrastructure likely generates broader impacts beyond ridership, including emissions reduction (40), retail activity, and crash risk or injury severity (41), which warrant further investigation. Fourth, the perception measures require careful interpretation. Place Pulse-derived scores capture general visual streetscape perceptions rather than cyclist-specific judgments, and the crowdsourced Mapillary imagery may retain heterogeneity associated with capture device, illumination, time of day, season, and contributor coverage despite spatial sampling and image-quality screening. Future research could strengthen external validity by collecting cyclist-specific ratings for a stratified subset of NYC street-view images and comparing them with the transferred Place Pulse scores across different street types, intersections, and bicycle-facility configurations, while independently sourced or standardized imagery could further assess sensitivity to image-source heterogeneity. Finally, our estimates primarily capture near to medium term effects. Over the longer term, bike lanes may influence residential location, business activity, and network learning, as well as who chooses to cycle. Understanding these dynamic effects will require longitudinal and cross-city analyses. Overall, these findings point to the value of integrating environmental psychology and urban design into transportation planning, recognizing that how streets are visually experienced may shape mobility choices alongside traditional engineering variables.

## Methods

### Study context and data

Our bicycle ridership data were obtained from Citi Bike’s publicly available system trip records, which provide trip-level information for the NYC bikeshare network beginning in 2013 (42). Each trip record includes trip start and end timestamps, origin and destination stations, and station identifiers, allowing the reconstruction of monthly OD travel flows across the network. Additional rider and bicycle attributes vary across years as the reporting system evolved; however, station names and locations remain sufficiently consistent to support longitudinal analysis of route demand over time. For this study, we focus on the period from January 2018 through December 2023, providing six years of observations spanning substantial growth in Citi Bike usage and geographic coverage. Annual Citi Bike trips increased from approximately 17.6 million in 2018 to 35.1 million in 2023, nearly doubling over the study period. By September 2023, the system had also expanded to approximately 2,000 stations, compared with about 1,000 stations in August 2020, reflecting substantial growth in its spatial coverage. Citi Bike is currently the largest bikeshare system in the United States, and the selected period also coincides with substantial expansion of NYC’s protected and painted bicycle-lane network and provides sufficient pretreatment and post-treatment observations for the staggered causal-inference framework.

Information on bicycle infrastructure was obtained from the New York City Open Data bicycle routes dataset, which provides a geospatial inventory of the city’s bike-lane network together with installation timing and facility classifications (43). Each bike-lane segment includes the month and year of implementation, route geometry, and facility class. Following the city’s coding convention, Facility Class I segments are classified as protected bike lanes, Facility Class II segments as painted bike lanes, and Facility Class III segments as shared-lane markings (sharrows). Because our research focuses on the causal ridership impacts of low-stress versus conventional cycling facilities, we center the analysis on Class I (protected) and Class II (painted) bike lanes, grouping sharrows (Class III) with Class II as conventional facilities.

Linking Citi Bike trip data with bicycle lane data begins by spatially integrating Citi Bike station locations with New York City’s bicycle lane network as of the end of 2023. Using the street network, shortest path bicycle routes are generated between each origin and destination pair using OSMnx-based routing, resulting in a total of 28,186 OD routes (refer to online supplementary Note 2). Each route is then classified based on the type of bicycle infrastructure it intersects, including protected Class I lanes, painted Class II lanes, or no lane. These OD routes are linked to Citi Bike trip records over the 2018 to 2023 period, yielding more than 90 million observed trips. This integration allows each route to be characterized by both its infrastructure exposure and its temporal ridership patterns. Among the constructed routes, 9,233 OD routes are classified as treated, meaning they intersect newly installed bicycle infrastructure (Class I/II) during the study period, while the remaining 18,953 serve as control routes. To assess sensitivity to the single-shortest-path representation, we conducted an additional multi-route analysis using a fixed random sample of 5,000 OD pairs. For each OD pair, we generated up to five plausible routes and retained alternatives no longer than 1.5 times the shortest-route length. Route probabilities were estimated using a Path-Size Logit formulation that accounts for both route length and overlap among alternative paths. We compared the deterministic shortest-path representation with the probability-weighted multi-route representation using protected-lane exposure, overall bicycle-infrastructure exposure, dominant infrastructure class, route overlap, and expected route length. Full specifications and results are reported in online supplementary Note 2.4.

Our data on community sociodemographic data was collected from the American Community Survey 2023 5-year estimates (44), which provide detailed information on over 329 million Americans, including population statistics, median household income, employed population, gender distribution, and ethnic composition at the CBG level (refer to Table S2 for description of the tables). Additionally, data on auto-oriented intersections per square mile, total activity density, and total road network density at the block group level were sourced from the Environmental Protection Agency’s (EPA) Smart Location Database (45). National Walkability Index score, which measures walkability at the CBG level, was also obtained from the same database (45).

### Quantifying human perception of the visual streetscape environment

To characterize general visual streetscape perception, we developed a computer-vision workflow combining street-level imagery from Mapillary (21) with the MIT Place Pulse 2.0 benchmark (22). The human-perception measures used in this study are derived from the previously published MIT Place Pulse 2.0 benchmark and secondary SVI; no new human participants were recruited and no new perception survey was administered as part of this study. Place Pulse 2.0 contains 110,988 street-view images from 56 cities in 28 countries and approximately 1.17 million pairwise comparisons from 81,630 participants across six perceptual dimensions: *beautiful*, *livable*, *safe*, *wealthy*, *boring*, and *depressing*. Image-level Q-scores derived from these pairwise judgments were used as supervised labels representing the relative perception of each image along a given dimension. Previous studies provide methodological precedent for transferring Place Pulse-derived perception models beyond their original imagery and geographic contexts. Zhang *et al.* (46) applied human-perception models to Beijing and Shanghai, Hou *et al.* (47) applied Place Pulse 2.0-pretrained models to crowdsourced Mapillary and KartaView imagery across 688 cities, and Galaktionova *et al.* (48) applied a Place Pulse-trained model to Mapillary imagery in Zagreb and compared the resulting scores with locally collected photographs and resident perceptions. Earlier Place Pulse research also included NYC imagery and demonstrated meaningful associations between image-derived urban perceptions and neighborhood-level outcomes (49).

For the NYC application, we retrieved georeferenced Mapillary imagery captured between 2018 and 2024 and spatially sampled locations at approximately 50-m intervals to reduce redundancy among closely spaced observations. A total of 12,664 sampling locations were identified across NYC, with four directional street-view images approximately 90 degrees apart used at each location, yielding 50,656 images for the perception analysis. Because Mapillary is crowdsourced, imagery can vary in capture device, camera configuration, time of day, illumination, season, and contributor behavior (50–52). We did not restrict imagery to a particular camera manufacturer/model or capture time, because such restrictions could substantially reduce spatial coverage. Instead, image selection emphasized visual suitability for streetscape assessment, including sufficient image clarity, illumination, visibility, and representation of the surrounding street environment. Images exhibiting severe blur, inadequate illumination, substantial obstruction, or insufficient representation of the street environment were excluded during the image-selection process.

For each image *i*, semantic scene composition was extracted using PSPNet with a ResNet-50 backbone (23). The model segments each image into 150 object categories, including built and natural streetscape elements such as buildings, roads, sidewalks, sky, trees, and vehicles. For each semantic category *k*, we calculated its image-level area ratio as:


(1) $$s_{ik}=\frac{1}{N_{i}}\sum_{\;p=1}^{N_{i}}1({\hat{y}}_{ip}=k),$$

where $N_{i}$ is the number of pixels in image *i*, and ${\hat{y}}_{ip}$ is the predicted semantic class of pixel *p*. The resulting vector $s_{i}$ summarizes the visual composition of each streetscape image. We then trained perception-specific support vector regression models with radial basis function kernels to predict image-level perception scores from the semantic composition features:


(2) $${\hat{q}}_{im}=f_{m}(s_{i})+\varepsilon_{i},$$

where ${\hat{q}}_{im}$ denotes the predicted score for perception dimension *m* in image *i*, and $f_{m}(\cdot )$ is the trained perception model. Separate models were estimated for beautiful, livable, safe, wealthy, boring, and depressing. Model performance was evaluated using cross-validation procedures, and detailed predictive accuracy statistics are reported in online supplementary Note 1 and Table S1. Predicted perception scores were spatially linked to street segments and subsequently associated with route origins and destinations for use in the OD-level causal framework.

To examine how objective built-environment conditions relate to perceived streetscape quality, we additionally estimated regression models in which each perception score was modeled as a function of neighborhood form and accessibility characteristics:


(3) $${\bar{q}}_{gm}=\alpha_{m}+X_{g}^{⊤}\beta_{m}+u_{g},$$

where ${\bar{q}}_{gm}$ is the average perception score for geographic unit *g*, and $X_{g}$ includes built-environment characteristics such as population density, road density, intersection density, walkability, activity density, and network accessibility. Full regression outputs and robustness checks are provided in online supplementary Note 2 (Tables S2–S7).

Finally, we computed Shapley Additive Explanation values to interpret the contribution of each built-environment feature to the perception model:


(4) $$f(x_{i})=\phi_{0}+\sum_{\;j=1}^{M}\phi_{ij},$$

where $\phi_{0}$ is the baseline prediction and $\phi_{ij}$ represents the contribution of feature *j* to the prediction for observation *i*. This analysis identifies the built-environment characteristics most strongly associated with variation in the predicted perception scores.

The perception scores used in the causal analysis were defined at the street-segment level and linked to Citi Bike OD routes through their route origins and destinations. To preserve causal ordering, only pretreatment perception scores were used. For treated routes, perception values were assigned from street segments observed prior to the installation month of the corresponding bicycle facility. For control routes, perception values were assigned using the matched pretreatment reference period. These origin-side and destination-side pretreatment perception measures were subsequently incorporated into the PSM framework and heterogeneous treatment-effect analysis. By integrating computer-vision-derived perception measures with quasi-experimental route-level inference, the framework evaluates whether bicycle-infrastructure effects vary across different perceived streetscape contexts.

### PSM for staggered OD route comparison

Before estimating treatment effects using the staggered DID framework, we refine the selection of control routes using PSM. Our matching framework differs from conventional station-level applications by defining the unit of analysis as the OD route. Treated routes are those exposed to a newly installed painted or protected bicycle lane, while control routes are untreated routes observed in the same staggered installation year. This staggered route-pairing design produces a more credible counterfactual set by jointly accounting for treatment timing, route demand, and the characteristics of both trip endpoints.

To estimate the probability that route *i* receives treatment, we fit a logistic regression model in which the treatment indicator is regressed on pretreatment route characteristics:


(5) $$\text{Pr}(T_{i}=1|X_{i})=\frac{\exp\;(\eta_{i})}{1+\exp\;(\eta_{i})}$$

where


(6) $$\eta_{i}=\beta_{0}+\beta_{1}\text{ln}({\text{BaselineTrips}}_{i})+\gamma_{y(i)}+\theta^{⊤}O_{i}+\delta^{⊤}D_{i}.$$

Here, $T_{i}=1$ indicates that route *i* is assigned to treatment, $\text{ln}({\text{BaselineTrips}}_{i})$ is the logarithm of pretreatment ridership, and $\gamma_{y(i)}$ denotes installation-year fixed effects that align routes within the same staggered rollout cohort. The vector $O_{i}$ contains origin-side covariates, while $D_{i}$ contains destination-side covariates.

The matching covariates were selected to capture the underlying factors associated with both bike-lane placement and cycling demand. These characteristics include: (i) built-environment variables sourced from the EPA’s Smart Location Database, including walkability, population density, road density, intersection density, activity density, and road-network accessibility, which jointly reflect connectivity, land-use intensity, and route attractiveness (45); (ii) sociodemographic variables obtained from the American Community Survey 2023 5-year estimates, including median household income, racial composition, and unemployment rates, which account for neighborhood economic conditions and potential differences in cycling participation (44); (iii) route-level demand, represented by baseline pretreatment ridership derived from Citi Bike trip records, ensuring that treated routes are compared with controls exhibiting similar initial cycling activity; and (iv) machine learning-derived streetscape perception measures from street-level imagery, including beautiful, boring, depressing, livable, safe, and wealthy, which capture how urban environments are visually experienced by potential riders and are spatially linked to route origins and destinations.

We implemented one-to-one nearest-neighbor PSM without replacement. To preserve temporal comparability under staggered treatment adoption, treated routes were matched only to control routes within the same assigned installation year. Within each year-specific stratum, matches were selected based on the nearest propensity score subject to a caliper of 0.02, ensuring that only sufficiently similar treated and control routes were paired. This procedure yields a matched analytical sample of 5,466 treated routes and 5,466 matched control routes, producing 10,932 route observations for the subsequent heterogeneous treatment analysis.

Following matching, we assess covariate balance using standardized mean differences (SMDs) and propensity score overlap diagnostics. The SMD for covariate *k* is defined as:


(7) $${\text{SMD}}_{k}=\frac{{\bar{x}}_{k,T}-{\bar{x}}_{k,C}}{\sqrt{\frac{s_{k,T}^{2}+s_{k,C}^{2}}{2}}}$$

where ${\bar{x}}_{k,T}$ and ${\bar{x}}_{k,C}$ are the matched sample means for treated and control routes, and $s_{k,T}^{2}$ and $s_{k,C}^{2}$ are the corresponding variances. An SMD below 0.10 for a given covariate suggests sufficient overlap between the treatment and control groups, indicating that the matching process has successfully minimized bias in the covariate distributions. This matching stage is critical to the identification strategy. By pairing treated routes with untreated routes that had similar pretreatment ridership, comparable endpoint contexts, and common treatment timing, the subsequent DID and CATE estimates rely on a substantially more credible “apples-to-apples” comparison between treated and untreated mobility corridors.

### DID analysis across bike lane type

Following the construction of matched treated and control route pairs using the PSM framework, we estimate the causal effect of bicycle infrastructure on ridership using a DID specification that distinguishes between painted and protected bike lanes. Because bicycle lanes were introduced at different times across corridors, this framework exploits staggered treatment timing while controlling for both route-specific and common temporal confounders. The model specification is defined as:


(8) $$\text{ln}(Y_{it})=\alpha +\beta_{0}W_{it}1(C_{i}=0)+\beta_{1}W_{it}1(C_{i}=1)+\mu_{i}+\lambda_{t}+\varepsilon_{it}$$

where $Y_{it}$ is the “number of bike trips +1” observed on OD route *i* in month *t*, allowing the logarithmic transformation to be applied when zero-trip observations occur. This semi-log transformation reduces right-skewness in ridership counts while preserving route-month observations with no recorded trips. The variable $W_{it}$ is a binary indicator equal to 1 if route *i* is exposed to bicycle infrastructure and the corresponding installation month has been reached, and 0 otherwise. Thus, $W_{it}$ switches from zero to one in the post-treatment period for treated routes, while remaining zero for untreated controls. The variable $C_{i}\in \{0,1\}$ denotes the type of bicycle lane treatment assigned to route *i*, where $C_{i}=0$ indicates a painted bike lane and $C_{i}=1$ indicates a protected bike lane. Therefore, $\beta_{0}$ captures the average treatment effect of painted bike lanes on logged ridership, while $\beta_{1}$ captures the corresponding effect of protected bike lanes. The term $\mu_{i}$ represents route-level fixed effects, absorbing all time-invariant differences across OD routes, including persistent variation in geography, built form, land use, and baseline cycling propensity. The term $\lambda_{t}$ denotes time fixed effects at the year-month level, capturing common shocks affecting all routes simultaneously, such as seasonality, weather fluctuations, fuel prices, and citywide mobility trends. Finally, $\varepsilon_{it}$ denotes the idiosyncratic error term. Standard errors are clustered at the route level to account for serial correlation within routes over time. This specification therefore enables a direct comparison of whether low-stress protected facilities generate larger ridership gains than conventional painted infrastructure.

### Event study analysis and placebo test

To assess the validity of the parallel trends assumption between OD routes receiving bicycle infrastructure treatment (painted or protected lanes) and comparable untreated routes, and to examine the temporal evolution of treatment effects before and after installation, we conduct an event study analysis. The presence of parallel pretreatment trends implies that, in the absence of treatment, ridership differences between treated and control routes would have remained stable over time, thereby strengthening the credibility of the staggered DID design. The event study specification is given as follows:


(9) $$\text{ln}(Y_{it})=\sum_{k\neq -1}\beta_{k}\left(1\{{\text{EventMonth}}_{it}=k\}\times Treated_{i}\right)+\mu_{i}+\varepsilon_{it}$$

where $Y_{it}$ is the “number of bike trips +1” observed on OD route *i* in month *t*. The variable $Treated_{i}$ is a binary indicator equal to 1 if route *i* eventually receives a bicycle lane treatment (painted or protected), and 0 otherwise. The indicator function $1\{{\text{EventMonth}}_{it}=k\}$ identifies the relative month *k* with respect to the treatment date, where k=0 denotes the month of installation, negative values represent pre-treatment periods, and positive values represent post-treatment periods. The omitted reference period is k=−1, corresponding to the month immediately preceding installation. Accordingly, each coefficient $\beta_{k}$ measures the difference in logged ridership between treated and control routes in relative month *k*, compared with the baseline month just before treatment. The term $\mu_{i}$ denotes route fixed effects, absorbing all time-invariant differences across OD routes such as geography, land-use structure, and baseline cycling propensity. The error term $\varepsilon_{it}$ captures idiosyncratic unexplained variation. Under the parallel trends assumption, coefficients for pretreatment periods (k<0) should be statistically indistinguishable from zero. Significant post-treatment coefficients (k≥0) indicate the emergence and persistence of treatment effects following bike-lane installation. This framework therefore provides a dynamic test of identification validity while revealing how ridership responses evolve after new bicycle infrastructure is introduced.

To test the hypothesis that changes in bikeshare ridership are driven by the installation of bicycle infrastructure rather than by spurious temporal trends, we conduct a placebo test for the staggered treatment framework. In this placebo design, we randomly shuffle the installation dates of treated bicycle lanes while preserving the observed set of treated routes and all route characteristics. Each treated route is therefore assigned a false installation month using the sklearn.utils.shuffle function in Scikit-learn. This procedure evaluates whether the estimated treatment effects could arise mechanically from unrelated time-varying shocks, residual selection bias, or chance correlations in the panel structure. If the main results were driven by such spurious factors rather than the actual timing of bike-lane deployment, then similarly strong effects would also be expected under randomly assigned treatment dates. Using this random reassignment process, we generate ten placebo datasets. For each placebo sample, we replicate the full empirical workflow: treated routes are paired with untreated routes using the same staggered PSM framework, after which the Difference-in-Differences model in Equation (4) is re-estimated using the placebo treatment timing.

### Heterogeneous treatment effects using a multi-arm causal forest

To examine whether the effectiveness of bicycle infrastructure varies systematically across neighborhood contexts, we estimate heterogeneous treatment effects using a multi-arm causal forest model. Whereas the staggered DID framework identifies average treatment effects, it implicitly summarizes impacts across all treated routes. In practice, however, behavioral responses to infrastructure are unlikely to be spatially uniform. The same bicycle lane may generate large ridership gains in one corridor but smaller effects in another depending on surrounding built form, socio-economic conditions, and perceived streetscape quality. To uncover this variation, we implement a doubly robust CausalForestDML estimator that jointly models treatment assignment and outcome dynamics while allowing flexible nonlinear interactions among route characteristics.

We construct the causal forest sample using the propensity-score-matched treated and control route pairs developed in the previous stage of the analysis. For each OD route *i*, the outcome variable is defined as the change in logged ridership between the post- and pretreatment periods:


(10) $$\Delta \text{ln}(Y_{i})=\text{ln}({\text{PostTrips}}_{i})-\text{ln}({\text{PreTrips}}_{i})$$

where the transformation captures proportional changes in route-level cycling activity while accommodating zero-trip observations. The treatment variable is multi-valued, such that $W_{i}\in \{0,1,2\}$, where $W_{i}=0$ denotes matched control routes, $W_{i}=1$ denotes routes exposed to painted bike lanes, and $W_{i}=2$ denotes routes exposed to protected bike lanes. The causal forest estimates the CATE:


(11) $$\tau_{k}(x)=E[Y_{i}(k)-Y_{i}(0)|X_{i}=x],\;k\in \{1,2\}$$

where $Y_{i}(k)$ denotes the potential outcome under treatment type *k*, $Y_{i}(0)$ is the potential outcome under no new infrastructure, and $X_{i}$ is a vector of pretreatment route characteristics. These covariates include baseline ridership, origin-side measures of walkability, population density, road density, intersection density, activity density, socio-economic composition, and machine learning-derived streetscape perceptions, together with analogous destination-side indicators.

Following the double machine learning framework, the CausalForestDML estimator uses random forests to account for outcome and treatment assignment processes, and then learns treatment effects from orthogonalized residual variation. This improves robustness to model misspecification in observational settings. The resulting model partitions the covariate space to maximize treatment-effect heterogeneity, allowing infrastructure impacts to vary flexibly across route contexts without requiring interaction terms to be specified a priori. We employ this framework for three reasons. First, it captures complex nonlinear relationships between perceived streetscapes and infrastructure effectiveness that conventional linear interaction models may overlook. Second, it complements the matched DID analysis by retaining all matched observations while uncovering within-sample heterogeneity. Third, it enables valid statistical inference for route-level treatment effects, allowing us to compare how protected and painted bike lanes perform across diverse urban environments. The resulting estimates form the basis of the heterogeneous effect analyses reported in Figure 3.

### Perception-based multivariate moderation of route-level treatment effects

To identify which perceived neighborhood characteristics explain the heterogeneity detected by the causal forest, we estimate separate multivariate moderation models for painted and protected bicycle lanes. In this stage, the route-level treatment effects predicted by the causal forest are used as dependent variables, allowing the perceptual correlates of treatment variation to be isolated. For each lane type l∈{painted,protected}, we estimate:


(12) $$\tau_{i}^{\left(l\right)}=\alpha^{\left(l\right)}+\sum_{k=1}^{6}\beta_{k}^{\left(l\right)}z_{ik}+\varepsilon_{i}$$

where $\tau_{i}^{\left(l\right)}$ denotes the estimated treatment effect for route *i* under lane type *l*, and $z_{ik}$ represents the standardized score of perception dimension *k*: beautiful, livable, safe, wealthy, boring, and depressing. All perception variables are standardized to mean zero and unit variance prior to estimation. Accordingly, each moderation coefficient $\beta_{k}^{\left(l\right)}$ can be interpreted as the change in the estimated treatment effect associated with a one standard deviation increase in perception dimension *k*, holding the remaining perceptions constant. The intercept $\alpha^{\left(l\right)}$ represents the expected treatment effect for lane type *l* in an average neighborhood where all standardized perceptions equal zero. Models are estimated separately for painted and protected infrastructure using ordinary least squares with heteroskedasticity-robust (HC3) standard errors.

### Predicted lane impacts across the perception distribution

To translate the moderation coefficients into substantively interpretable treatment effects, we compute predicted lane impacts across the observed distribution of each perception dimension while holding all remaining perception variables at their mean values. For lane type *l* and perception variable *k*, the predicted treatment effect is:


(13) $${\hat{\tau }}^{\left(l\right)}(z_{k})={\hat{\alpha }}^{\left(l\right)}+{\hat{\beta }}_{k}^{\left(l\right)}z_{k}$$

where $z_{k}$ denotes the standardized perception score, such that $z_{k}=0$ corresponds to the citywide average, positive values indicate neighborhoods perceived more strongly along that dimension, and negative values indicate neighborhoods perceived less strongly. This procedure traces how expected ridership gains vary continuously across the full empirical range of perceived streetscape conditions. To aid interpretation, each census block group is further classified according to whether the standardized perception score is negative $\left(z_{k}<-1\right)$, neutral $\left(-1\leq z_{k}\leq 1\right)$, or positive $\left(z_{k}>1\right)$, yielding six spatial categories that jointly summarize lane advantage and contextual intensity. By comparing predicted profiles for painted and protected lanes, the analysis reveals where lower-cost painted infrastructure may be sufficient and where physically separated facilities are likely to generate substantially larger returns.

### Street-level scenario predictions for untreated candidate corridors

To characterize the street-level scenarios using a reproducible perceptual typology, candidate segments were classified along two dimensions: general visually perceived safety and neighborhood soundness. The neighborhood-soundness dimension was adapted from the Neighborhood Soundness Index (NSI) proposed by Wang *et al.* (53), which combines multiple human-perception dimensions into an overall measure of perceived neighborhood quality. Because visually perceived safety was used separately as the first classification dimension, we constructed a safety-excluded NSI using beautiful, livable, wealthy, non-boring, and non-depressing perceptions, with equal weight assigned to each component. The resulting NSI and the visually perceived safety score were standardized relative to the NYC-wide distribution. Values above zero were classified as above average and values at or below zero as at or below average. This yielded four contextual combinations: higher safety–higher NSI, higher safety–lower NSI, lower safety–higher NSI, and lower safety–lower NSI. One representative corridor was selected from each class based on these observed perception characteristics, independently of the subsequent model-implied lane-scenario predictions. Full classification criteria, standardized scores, and corridor assignments are reported in online supplementary Note 3.5. We then apply the estimated moderation model to selected untreated or conventionally treated corridors to generate corridor-specific planning scenarios. For each street segment *s*, the expected treatment effect of lane type *l* is computed as:


(14) $${\hat{\tau }}_{s}^{\left(l\right)}={\hat{\alpha }}^{\left(l\right)}+\sum_{k=1}^{6}{\hat{\beta }}_{k}^{\left(l\right)}z_{sk}$$

where $z_{sk}$ is the standardized perception score of segment *s*. This prediction framework converts citywide heterogeneous treatment estimates into interpretable street-level expectations, allowing direct comparison of painted and protected facilities under diverse real-world environments. These scenario analyses are not used for causal identification. Rather, they serve as policy translation tools that illustrate how estimated treatment-response functions may inform future bicycle infrastructure prioritization.

## Supplementary Material

pgag354_Supplementary_Data

## Data Availability

Citi Bike trip data used in this study are publicly available and can be downloaded from the Citi Bike system data portal at https://citibikenyc.com/system-data. The New York City bicycle lane network data are publicly available from the NYC Open Data platform at https://data.cityofnewyork.us/dataset/New-York-City-Bike-Routes-Map-/9e2b-mctv. Other data including the American Community Survey is available for download at https://data.census.gov/, and Tiger shapefiles can be downloaded from the US Census Bureau https://www.census.gov/geographies/mapping-files/time-series/geo/tiger-line-file.html.

## References

[1] Schneider RJ . 2020. United States pedestrian fatality trends, 1977 to 2016. Transp Res Rec. 2674:1069–1083.

[2] Buehler R, Pucher J. 2017. Trends in walking and cycling safety: recent evidence from high-income countries, with a focus on the United States and Germany. Am J Public Health. 107:281–287. 10.2105/AJPH.2016.303546 PMC5227927 27997241

[3] De Silva MM, Baddewela SWYBPK, Sivakumar T, Nakayama T. 2025. Promoting sustainable mobility by addressing inequities in electric bike-sharing systems. J South Asian Logist Transp. 5:117–135.

[4] Chahine R, Duarte J, Gkritza K. 2025. Effect of protected bike lanes on bike-sharing ridership: a New York City case study. J Transp Geogr. 123:Article 104147.

[5] Ito K, Bansal P, Biljecki F. 2024. Examining the causal impacts of the built environment on cycling activities using time-series street view imagery. Transp Res Part A Policy Pract. 190:Article 104286.

[6] Moran M, Salman M, Yabe T. 2026. Heterogeneous impacts of protected bike lanes on bikeshare behavior across demographic groups in New York. NPJ Sustain Mobil Transp. 3:39.

[7] Cervero R, Kockelman K. 1997. Travel demand and the 3Ds: density, diversity, and design. Transp Res D Transp Environ. 2:199–219.

[8] Hwang U, Mokhtarian PL, Koo BW, Guhathakurta S. 2026. Perceived streetscape quality and bike lane effectiveness: a computer vision approach. Transp Res D Transp Environ. 154:Article 105248.

[9] Harvey C, Aultman-Hall L, Hurley SE, Troy A. 2015. Effects of skeletal streetscape design on perceived safety. Landsc Urban Plan. 142:18–28.

[10] Sugiyama T, Neuhaus M, Cole R, Giles-Corti B, Owen N. 2012. Destination and route attributes associated with adults’ walking: a review. Med Sci Sports Exerc. 44:1275–1286. 10.1249/MSS.0b013e318247d286 22217568

[11] Zhao B, Deng Y, Luo L, Deng M, Yang X. 2025. Preferred streets: assessing the impact of the street environment on cycling behaviors using the geographically weighted regression. Transportation. 52:1485–1511.

[12] Ferenchak NN, Marshall WE. 2025. The link between low-stress bicycle facilities and bicycle commuting. Nat Cities. 2:555–559.

[13] Berghoefer FL, Vollrath M. 2022. Cyclists’ perception of cycling infrastructure—a repertory grid approach. Transp Res Part F Traffic Psychol Behav. 87:249–263.

[14] Friel D, Wachholz S. 2025. Cyclists’ perception of cycling infrastructure: the relationship between safety, comfort, and comprehensibility. Transp Res Part F Traffic Psychol Behav. 115:Article 103367.

[15] De Silva MM, Clark C, Nakayama T, Yabe T. 2025. Causal spillover effects of electric vehicle charging station placement on local businesses: a staggered adoption study [preprint], arXiv, arXiv:2511.19507, https://arxiv.org/abs/2511.19507.

[16] Zheng Y, Keith DR, Wang S, Diao M, Zhao J. 2024. Effects of electric vehicle charging stations on the economic vitality of local businesses. Nat Commun. 15:1–11. 10.1038/s41467-024-51554-9 PMC11375121 39231925

[17] Broach J, Dill J, Gliebe J. 2012. Where do cyclists ride? A route choice model developed with revealed preference GPS data. Transp Res Part A Policy Pract. 46:1730–1740.

[18] Kahneman D, Tversky A. 1979. Prospect theory: an analysis of decision under risk. Econometrica. 47:263–291.

[19] Ulrich RS . 1983. Aesthetic and affective response to natural environment. Hum Behav Environ. 6:85–125.

[20] Ulrich RS , et al 1991. Stress recovery during exposure to natural and urban environments. J Environ Psychol. 11:201–230.

[21] Mapillary . 2025. Mapillary API documentation. https://www.mapillary.com/developer/api-documentation, Accessed 2025.

[22] MIT Media Lab . 2016. Place pulse. https://www.media.mit.edu/projects/place-pulse-1/overview/, Accessed 2025.

[23] Yang C, Guo H. 2022. A method of image semantic segmentation based on PSPNet. Math Probl Eng. 2022:Article 8958154.

[24] Ewing R, Dumbaugh E. 2009. The built environment and traffic safety: a review of empirical evidence. J Plan Lit. 23:347–367.

[25] Ewing R , et al Measuring urban design: metrics for livable places. Vol. 200. Island Press, 2013.

[26] Athey S., Wager S.. 2019. Estimating treatment effects with causal forests: an application. Obs Stud. 5:37–51.

[27] Ye Y, Zhong C, Suel E. 2024. Unpacking the perceived cycling safety of road environment using street view imagery and cycle accident data. Accid Anal Prev. 205:Article 107677. 10.1016/j.aap.2024.107677 38924963

[28] Dill J, McNeil N. 2016. Revisiting the four types of cyclists: findings from a national survey. Transp Res Rec. 2587:90–99.

[29] Juarez RNR, Grigolon AB, Madureira AM. 2023. Cyclists’ perception of streetscape and its influence on route choice: a pilot study with a mixed-methods approach. Transp Res Part F Traffic Psychol Behav. 99:374–388.

[30] Sanders RL . 2015. Perceived traffic risk for cyclists: the impact of near miss and collision experiences. Accid Anal Prev. 75:26–34. 10.1016/j.aap.2014.11.004 25460088

[31] Larkin A, Gu X, Chen L, Hystad P. 2021. Predicting perceptions of the built environment using GIS, satellite and street view image approaches. Landsc Urban Plan. 216:Article 104257. 10.1016/j.landurbplan.2021.104257 PMC8494182 34629575

[32] Friel D , et al 2023. Cyclists’ perceived safety on intersections and roundabouts—a qualitative bicycle simulator study. J Safety Res. 87:143–156. 10.1016/j.jsr.2023.09.012 38081689

[33] Winters M, Davidson G, Kao D, Teschke K. 2011. Motivators and deterrents of bicycling: comparing influences on decisions to ride. Transportation. 38:153–168.

[34] Guo Y, He SY. 2021. The role of objective and perceived built environments in affecting dockless bike-sharing as a feeder mode choice of metro commuting. Transp Res Part A Policy Pract. 149:377–396.

[35] Ma L, Cao J. 2019. How perceptions mediate the effects of the built environment on travel behavior? Transportation. 46:175–197.

[36] Furth PG, Mekuria MC, Nixon H. 2016. Network connectivity for low-stress bicycling. Transp Res Rec. 2587:41–49.

[37] Ma L, Dill J. 2017. Do people’s perceptions of neighborhood bikeability match “reality”? J Transp Land Use. 10:291–308.

[38] National Association of City Transportation Officials . 2025. Urban bikeway design guide: designing bikeways for all ages and abilities. https://nacto.org/publication/urban-bikeway-design-guide/designing-bikeways-for-all-ages-and-abilities/, Accessed 2026.

[39] Willis DP, Manaugh K, El-Geneidy A. 2015. Cycling under influence: summarizing the influence of perceptions, attitudes, habits, and social environments on cycling for transportation. Int J Sustain Transp. 9:565–579.

[40] US Government . 2022. Emissions of carbon dioxide in the transportation sector. Congressional Budget Office. Technical Report.

[41] Pucher J, Buehler R. 2017. Cycling towards a more sustainable transport future. Transp Rev. 37:689–694.

[42] Citi Bike NYC . 2024. Citi bike system data. Available at: https://citibikenyc.com/system-data.

[43] New York City Department of Transportation . 2024. New York city bike routes map. Available at: https://data.cityofnewyork.us/dataset/New-York-City-Bike-Routes-Map-/9e2b-mctv.

[44] U.S. Census Bureau . 2022. American community survey (ACS). Available at: https://www.census.gov/programs-surveys/acs.

[45] U.S. Environmental Protection Agency . 2022. Smart location database. Available at: https://www.epa.gov/smartgrowth/smart-location-mapping.

[46] Zhang F , et al 2018. Measuring human perceptions of a large-scale urban region using machine learning. Landsc Urban Plan. 180:148–160.

[47] Hou Y , et al 2024. Global Streetscapes—a comprehensive dataset of 10 million street-level images across 688 cities for urban science and analytics. ISPRS J Photogramm Remote Sens. 215:216–238.

[48] Galaktionova A, Istrate A-L, Sonet UN, Carroll P, Babojelić K. 2026. Mapping vitality–safety tensions with streetscape scores in Zagreb. J Urban Des. 1–19. 10.1080/13574809.2026.2655850.

[49] Salesses P, Schechtner K, Hidalgo CA. 2013. The collaborative image of the city: mapping the inequality of urban perception. PLoS One. 8:Article e68400. 10.1371/journal.pone.0068400 PMC3722224 23894301

[50] Ma D, Fan H, Li W, Ding X. 2020. The state of mapillary: an exploratory analysis. ISPRS Int J Geoinf. 9:10.

[51] Mahabir R, Schuchard R, Crooks A, Croitoru A, Stefanidis A. 2020. Crowdsourcing street view imagery: a comparison of mapillary and OpenStreetCam. ISPRS Int J Geoinf. 9:341.

[52] Biljecki F, Ito K. 2021. Street view imagery in urban analytics and GIS: a review. Landsc Urban Plan. 215:Article 104217.

[53] Wang S , et al 2025. Reducing the social inequity of neighborhood visual environment in Los Angeles through computer vision and multi-model machine learning. Sustain Cities Soc. 119:Article 106062. 10.1016/j.scs.2024.106062 PMC11781154 39896741

