# Supplementary material for: Streetscape perception conditions the causal impacts of low-stress bicycle infrastructure
Source: PNAS Nexus. 2026 Nov 10;5(11):pgag354. doi: 10.1093/pnasnexus/pgag354 (PMC13756087; doi:10.1093/pnasnexus/pgag354)
Supplement: Supplementary file 1 [file pgag354_supplementary_data.pdf]

Supplementary Information for

Streetscape perception conditions the causal impacts of low-stress bicycle infrastructure

M. Mavin De Silva[1,2,4,*], Siqin Wang[3,*], Ashani Piyatilake[1], Sijian Tian[4], Santiago Garcia Gabilondo[4], Tadachika Nakayama[1,*]

[1]Extreme Energy-Density Research Institute, Nagaoka University of Technology, Nagaoka, Niigata 940-2188, Japan

[2]Department of Transport Management & Logistics Engineering, Faculty of Engineering, University of Moratuwa, Katubedda 10400, Sri Lanka

[3]Spatial Sciences Institute, Dornsife College of Letters, Arts and Sciences, University of Southern California, Los Angeles, CA, United States

[4]Center for Spatial Information Science, University of Tokyo, Komaba, Tokyo, Japan

*Corresponding authors: `mavinds@uom.lk, siqinwan@usc.edu, nky15@vos.nagaokaut.ac.jp`

Supplementary Notes

1 Data 4
- 1.1 Citi Bike Data 4
  - 1.1.1 Temporal growth and spatial coverage of the Citi Bike network . . 4
- 1.2 New York City Bike Lanes 6
- 1.3 Street View Images and Spatial AI Models 8
  - 1.3.1 Pairwise relationships among human-perception dimensions 10
- 1.4 American Community Survey and Smart Location Detailed Tables 11

2 Empirical Strategy and Causal Identification 11
- 2.1 Construction of OD Routes and Treatment Assignment 12
- 2.2 Propensity Score Matching 13
- 2.3 Difference-in-differences Analysis Across Bike Lane Type 17
- 2.4 Destination-specific route information and alternative-route sensitivity . . 18
  - 2.4.1 Destination-specific information retained by the OD-pair representation 19
  - 2.4.2 Sensitivity to plausible alternative-route assignments 20

3 Heterogeneous Treatment Effects and Perception-Based Mechanisms 23
- 3.1 Heterogeneous Treatment Effects Using a Multi-Arm Causal Forest . . . 23
- 3.2 Perception based Moderation of Treatment Effects 24
  - 3.2.1 Univariate Moderation Analysis 24
- 3.3 Spatial autocorrelation of perception-conditioned treatment-effect patterns 27
  - 3.3.1 Global spatial autocorrelation 27
  - 3.3.2 Local spatial association 28

3.3.3 Interpretation in relation to Figure 3c 29
3.4 Predicted Treatment Effects Across the Perception Distribution 30
3.5 Safety–neighborhood soundness classification of street-level scenarios . . . 30

List of Figures

S1 Spatial coverage of the Citi Bike flow network used in the analysis 5
S2 Annual Citi Bike trip volumes for matched OD routes, 2018-2023 5
S3 Spatial Distribution of Origin–Destination (OD) Bikeshare Stations in New York City (NYC). 7
S4 Spatial Patterns of the Six Perceived Neighborhood Visual Environment Indices at the Census Block Group Level. 9
S5 **Destination-specific variation in protected-lane exposure among routes sharing the same origin station.** Distribution of the within-origin range in protected-lane route exposure across origin stations represented by at least two sampled OD pairs. The range is calculated as the difference between the maximum and minimum protected-lane exposure among destination-specific routes sharing the same origin. The median within-origin range is 0.336, indicating substantial destination-specific variation in protected-lane environments encountered by trips departing from the same Citi Bike station. 20
S6 Sensitivity of protected-lane exposure to alternative-route assignment. . . 22
S7 Forest Plot of Moderation Effeccts on Treatment Effects 25
S8 Incremental Effects of Perceived Streetscape Characteristics on Bicycle Infrastructure Impacts 31

List of Tables

S1 Model Performance Metrics Across Perception Dimensions 10
S2 Detailed Tables from American Community Survey (ACS) and Smart Location Database (SDL) 12
S3 Logistic Regression Predicting Treatment Assignment 14
S4 Descriptive Statistics for Bike Lane Treated and Matched Control OD Routes 15
S5 Effect of Treatment on Bike Trips for both Protected and Painted Bike Lanes 18
S6 Destination-specific information retained by the OD-pair representation. 20
S7 Sensitivity of infrastructure exposure to alternative-route assignment. . . 22
S8 Univariate Perception based Moderation of Route Level Treatment Effects 25
S9 Multivariate Perception based Moderation of Route Level Treatment Effects 27
S10 Global Moran's I for perception-conditioned relative advantage surfaces. 28
S11 Local Moran's I cluster classifications. 29
S12 Safety–neighborhood soundness classification of the four street-level scenarios shown in Figure 4. 32

1 Data

1.1 Citi Bike Data

Citi Bike trip data are publicly available for all trips in the system's history, beginning in 2013, and can be accessed from the official system data portal at https://citibikenyc.com/system-data. Each trip record includes the trip start and end timestamps, origin and destination stations, and station identifiers, enabling the reconstruction of origin–destination (OD) travel flows across the network. Additional rider or bicycle specific attributes have changed over time as the reporting system evolved. For example, rider demographic information such as gender and age was included in earlier years but later discontinued, while other attributes such as bike electrification status were introduced more recently. Despite these changes, station names and geographic locations have remained largely consistent across years, allowing reliable longitudinal analysis of network usage. By aggregating trips at the station level, the spatial extent of the bikeshare system can be mapped and tracked over time.

1.1.1 Temporal growth and spatial coverage of the Citi Bike network

Our analysis covers Citi Bike origin–destination (OD) trips from 2018 through 2023, a six-year period characterized by substantial changes in both ridership and the spatial extent of bike-share activity across New York City. To provide context for the analytical sample, we summarize both the geographic distribution of observed Citi Bike flows and the temporal evolution of trip volumes during the study period.

Supplementary **Figure S1** illustrates the spatial coverage of the Citi Bike OD network represented in our data. The left panel shows network links associated with positive Citi Bike flows, with higher-intensity links indicating corridors carrying larger volumes of observed trips. The right panel overlays the same flow network with the geographic boundary enclosing the observed Citi Bike network. The resulting spatial footprint shows extensive coverage across Manhattan and substantial portions of Brooklyn, Queens, and the Bronx, while also illustrating the uneven concentration of bicycle flows within the broader network. This spatial representation provides the geographic context for the OD-route construction and subsequent bicycle-infrastructure exposure analysis.

Supplementary **Figure S2** presents the annual number of Citi Bike trips associated with the matched OD routes retained in the causal analysis. Trip volumes increase from approximately 1.4 million trips in 2018 to 1.8 million in 2019, followed by a temporary decline to approximately 1.6 million trips in 2020. Matched-route ridership subsequently increased substantially, reaching approximately 2.6 million trips in 2021, 3.3 million in 2022, and 4.5 million in 2023. Thus, the matched analytical sample reflects the broader temporal pattern of disruption around the COVID-19 period followed by substantial growth in bicycle-share use.

These temporal changes occurred alongside the continued expansion of the Citi Bike system. At the system-wide level, Citi Bike recorded more than 44 million trips in 2023, more than twice its 2019 ridership and the highest annual ridership recorded by the system during our study period. Importantly, Supplementary Figure S2 reports trips only for the matched OD routes included in our causal analysis and therefore represents a subset of total system-wide ridership. Together, Supplementary Figures S1–S2 provide spatial and temporal context for the staggered treatment analysis by showing the geographic extent

of the OD network and the substantial growth in cycling activity over the 2018–2023 study period.

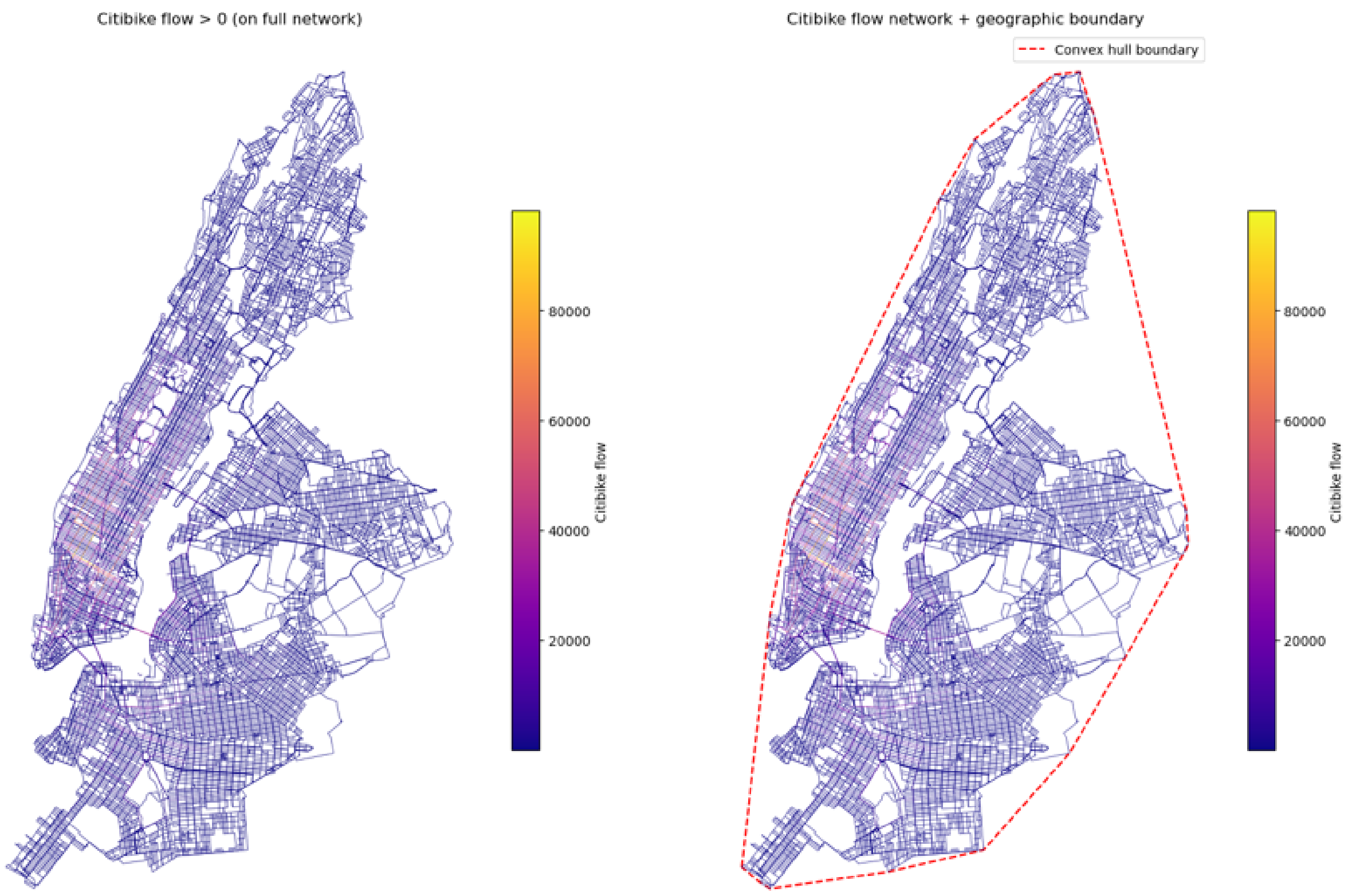

Figure S1: Spatial coverage of the Citi Bike flow network used in the analysis

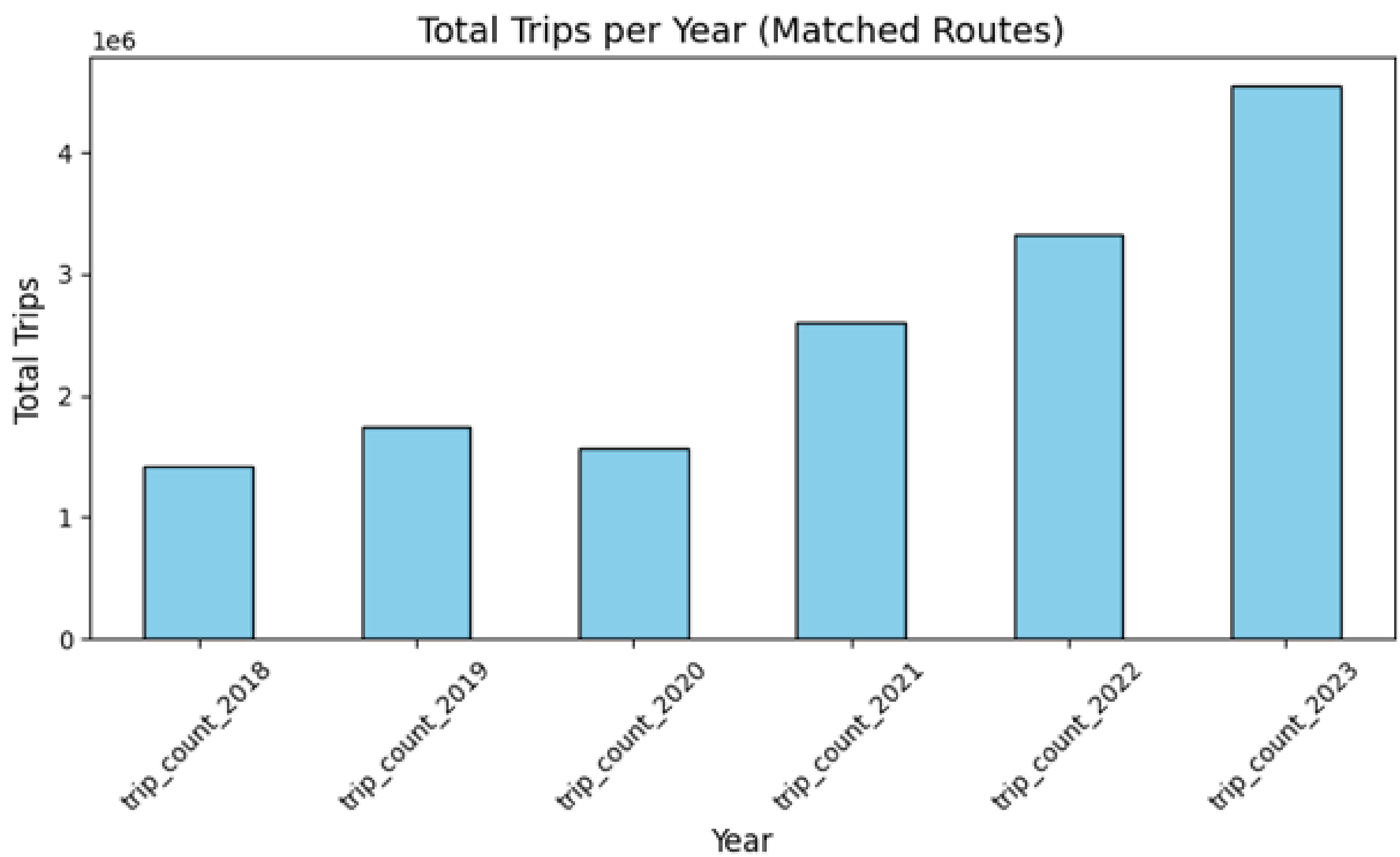

Figure S2: Annual Citi Bike trip volumes for matched OD routes, 2018-2023

1.2 New York City Bike Lanes

Information on bicycle infrastructure was obtained from the New York City Open Data bicycle routes dataset, which provides a comprehensive geospatial inventory of the city's bike-lane network and is available at https://data.cityofnewyork.us/dataset/New-York-City-Bike-Routes-Map-/9e2b-mctv. The dataset includes detailed attributes for each bike-lane segment, including route geometry, installation month and year, and facility classification. These data can be readily analyzed within GIS environments to reconstruct the spatial and temporal evolution of the bicycle network. Following the city's classification scheme, Facility Class I segments correspond to protected bike lanes, Facility Class II segments to painted bike lanes, and Facility Class III segments to shared-lane markings (sharrows). Because this study focuses on the causal impacts of low-stress versus conventional cycling infrastructure, the analysis centers on Class I and Class II facilities, with sharrows grouped together with Class II as conventional treatments. The dataset captures the progressive expansion of bicycle infrastructure across New York City over the study period and provides the temporal information necessary to link route exposure with the staggered rollout of bike lanes. **Figure S3** shows the selected areas in New York City by illustrating the distribution of bike stations and their adjacent bike lanes across each borough.

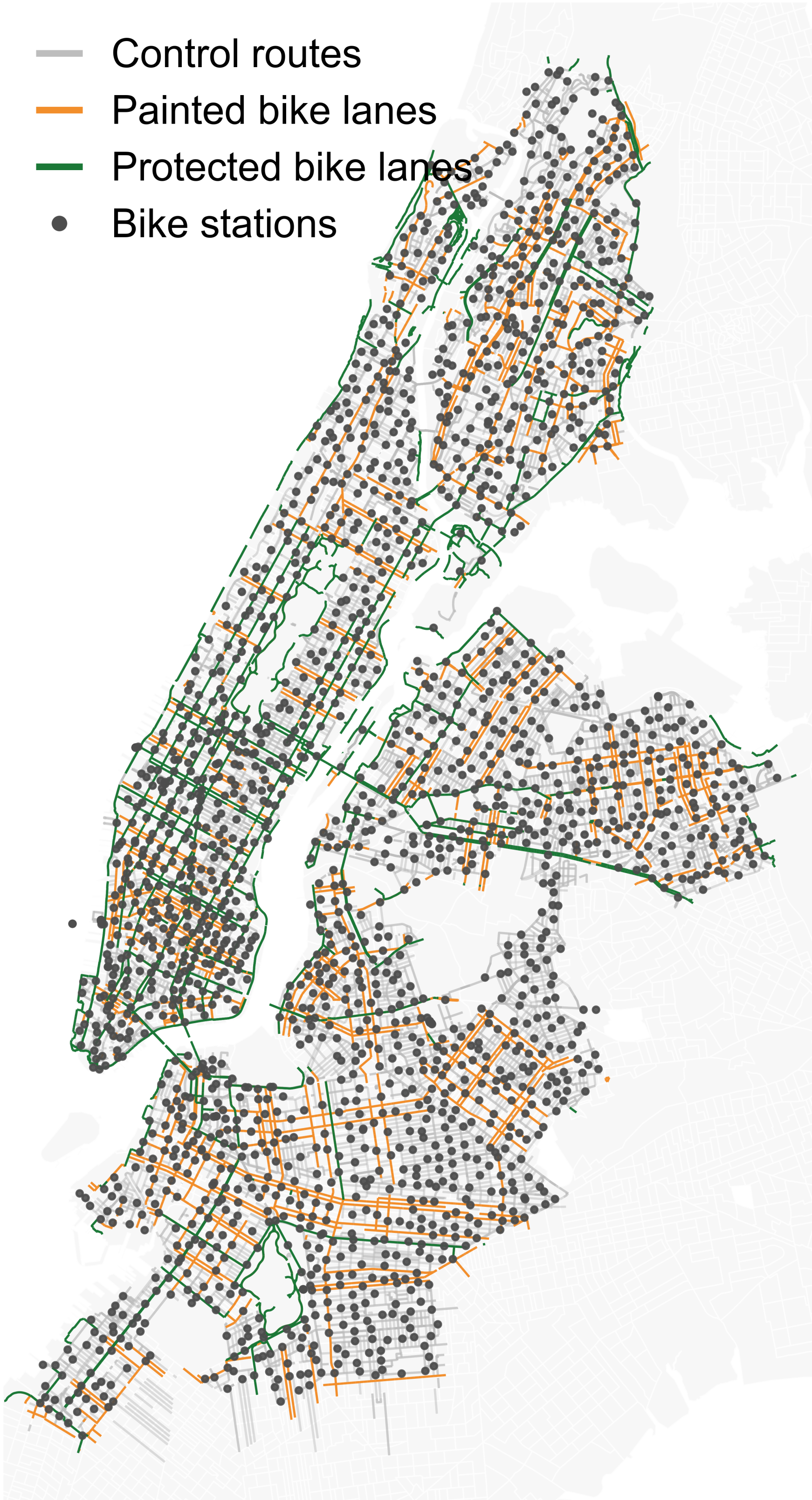

Figure S3: Spatial Distribution of Origin–Destination (OD) Bikeshare Stations in New York City (NYC).

1.3 Street View Images and Spatial AI Models

Street view imageries (SVI) were collected using the Mapillary API (v4), which provides access to georeferenced images contributed voluntarily through the crowdsourcing platform[3]. Mapillary SVI are acquired using diverse devices and mobile platforms, including smartphones, digital cameras, and vehicle-mounted cameras, yielding high-resolution visual data that are well suited for analyzing human perception on urban environment. A key advantage of Mapillary is its dense spatial coverage, with neighboring image capture locations separated by a median distance of approximately 1.8 m [3]. However, such high sampling density can introduce substantial image overlap and redundancy between adjacent observations. To reduce spatial duplication while maintaining representative urban coverage, we implemented a systematic sampling strategy at 50-m intervals, following the early studies [8]. This produced 50,656 SVI captured in 12,664 sampling locations across New York City, primarily concentrated in Manhattan, with additional coverage extending into Brooklyn, Queens, and Bronx.

We use the Place Pulse 'safe' score as a measure of general visually perceived streetscape safety rather than cyclist-specific safety. Cyclist-specific safety perceptions may place greater weight on traffic speed and volume, physical separation, intersection conditions, road width, and cycling-facility design than general visual judgments of streetscape safety. Previous cycling research nevertheless shows that perceived safety, separation from motor traffic, aesthetic quality, and other visible street characteristics influence cycling decisions and route choice [9, 1, 5, 10]. We therefore interpret the Place Pulse measures as contextual characteristics of the visual environment through which cyclists travel, rather than direct measurements of cyclists' own safety perceptions.

Using the collected SVI, we quantified six dimensions of perceived urban environment, i.e., feeling safe, wealthy, beautiful, livable, boring and depressing, through a systematic spatial AI framework trained on the MIT Place Pulse benchmark dataset (2016). The MIT Place Pulse project provides large-scale crowdsourced evaluations of urban scenes in 56 global cities, offering a robust reference for modelling human perception to the urban environment. For each training image, perceptual ratings were represented using Q-scores[4], a comparative metric derived from pairwise crowd judgements that captures the relative positivity of an image for a given perceptual attribute. These scores were normalized to a 0–10 scale, where higher values indicate stronger positive perception[8]. To characterize the visual composition of each street scene, we applied a PSPNet semantic segmentation model built on a ResNet-50 backbone[11]. This deep learning framework decomposed each image into 150 semantic object classes, including buildings, roads, vegetation, sky, and other urban elements, and quantified the proportional visual coverage of each category within the scene(**Figure S4**). These object-level composition metrics provided a structured representation of urban visual environments suitable for predictive modelling. We then integrated the semantic composition features with perceptual Q-scores to train a Support Vector Machine (SVM) model using a Radial Basis Function (RBF) kernel[2]. Model parameters were optimized through cross-validation to maximize predictive performance and generalizability. **Table S1** shows the modelling performance of six machine learning models by taking the six perceived visual environment indices as the dependent variable. The trained framework was subsequently applied to the full SVI dataset to generate spatially explicit estimates of six perceptual dimensions, enabling large-scale assessment of how urban built environments are visually experienced.

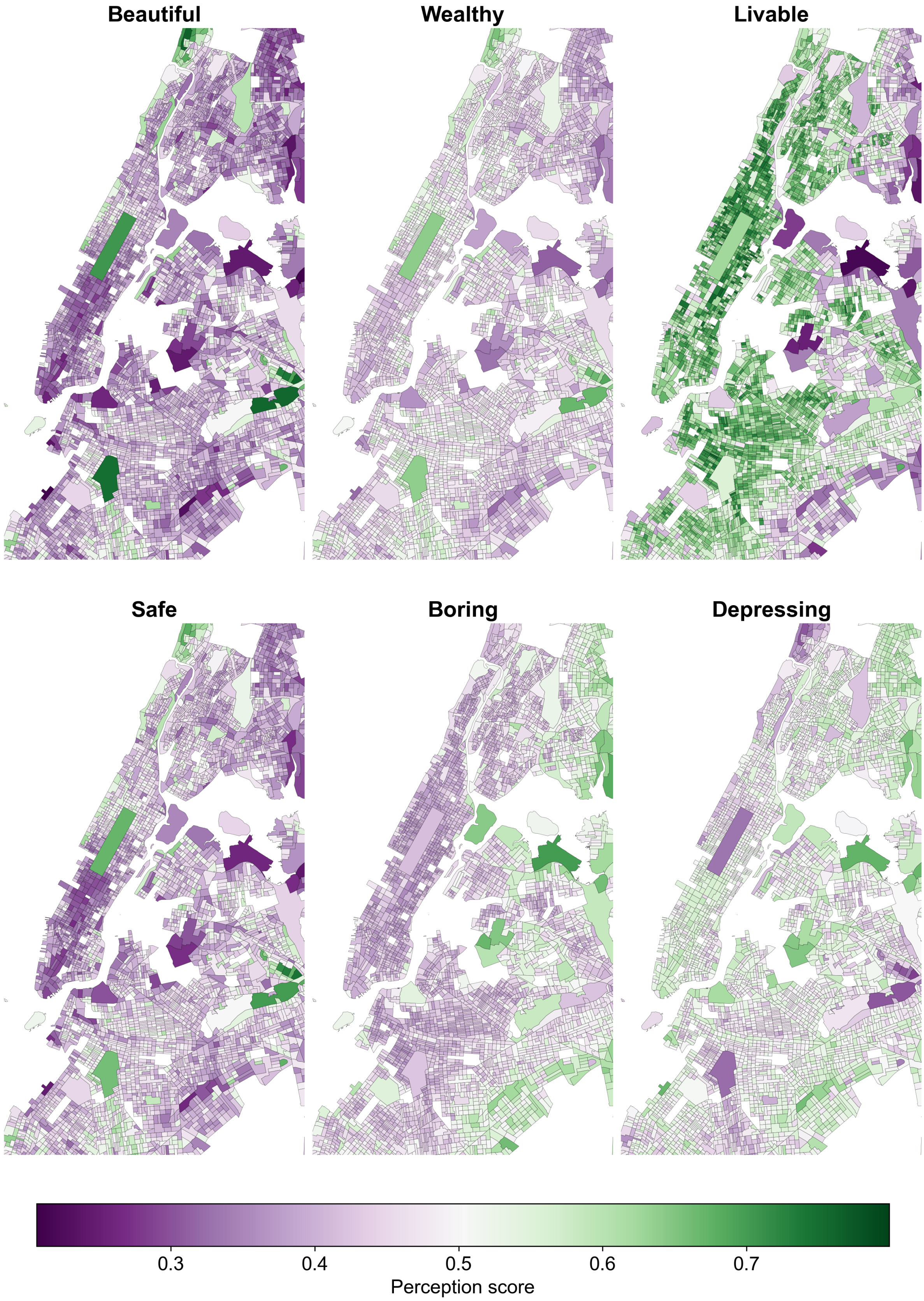

Figure S4: Spatial Patterns of the Six Perceived Neighborhood Visual Environment Indices at the Census Block Group Level.

Table S1: Model Performance Metrics Across Perception Dimensions

Metric	Beautiful	Wealth	Liveable	Safe	Boring	Depressive
MAE	0.055	0.045	0.072	0.059	0.041	0.044
MSE	0.005	0.003	0.008	0.006	0.003	0.003
RMSE	0.071	0.058	0.090	0.075	0.052	0.056
R-squared	0.519	0.425	0.365	0.438	0.400	0.471

1.3.1 Pairwise relationships among human-perception dimensions

The six human-perception measures used in this study beautiful, livability, safe, wealthy, boring, and depressing represent distinct dimensions of visual streetscape perception. Because these dimensions are derived from the same underlying visual environment, however, they need not be statistically independent. We therefore examined their pairwise relationships across the NYC perception observations used in the spatial analysis.

For observations with complete information across all six perception dimensions ($N =$ 12,662), we calculated both Pearson product–moment correlations and Spearman rank correlations. Pearson correlations quantify the strength of the linear association between two perception scores,

$$r_{XY} = \frac{\sum_{i=1}^{N}(X_i - \bar{X})(Y_i - \bar{Y})}{\sqrt{\sum_{i=1}^{N}(X_i - \bar{X})^2}\sqrt{\sum_{i=1}^{N}(Y_i - \bar{Y})^2}}, \tag{1}$$

whereas Spearman correlations quantify the corresponding monotonic association based on the ranks of the observations. Reporting both measures allows us to assess whether the observed relationships depend strongly on linearity or are also preserved in the rank ordering of street contexts.

The correlation analysis reveals substantial, but non-uniform, empirical overlap among the six perception dimensions. Particularly strong positive relationships are observed among beautiful, safe, and wealthy perceptions. Perceived beauty and wealth are strongly correlated ($r = 0.975$, $\rho = 0.969$), indicating that NYC streets perceived as more aesthetically attractive also tend to be perceived as more affluent. Perceived safety and wealth exhibit a similarly strong relationship ($r = 0.987$, $\rho = 0.982$), while beauty and perceived safety are also strongly positively associated ($r = 0.985$).

Livability does not exhibit an identical relationship with these positive-valence perceptions. For example, the association between livability and wealth is positive but smaller ($r = 0.661$) than the beauty–wealth or safety–wealth relationships. This supports treating livability as a separate perceptual dimension rather than interpreting it as synonymous with either aesthetic attractiveness or perceived affluence.

The relationships involving negatively valenced perceptions also reveal important distinctions. Livability and boringness exhibit a very strong inverse association ($r = -0.975$, $\rho = -0.974$), indicating that locations perceived as more livable generally tend to be perceived as less boring. This strong inverse relationship nevertheless does not imply that the two variables constitute the same construct with opposite coding. Livability captures a positive judgment about the perceived suitability or quality of the street environment, whereas boringness captures a judgment of visual monotony or lack of stimulation. Their strong empirical relationship in NYC therefore reflects covariance in how streets are perceived rather than definitional equivalence.

Boringness and depressingness are positively associated ($r = 0.653$, $\rho = 0.648$), but considerably less strongly than the livability–boring relationship. Thus, a street that appears visually unstimulating does not necessarily receive an equally strong depressing perception score. By contrast, perceived wealth and depressingness exhibit a particularly strong inverse association ($r = -0.993$), indicating that streets perceived as affluent are very rarely perceived as depressing in the NYC sample.

Taken together, these relationships demonstrate that the six perception dimensions are neither statistically independent nor interchangeable. Some dimensions share substantial empirical variation—particularly beauty, safety, and wealth—whereas other relationships are appreciably weaker. Accordingly, the perception measures are interpreted as overlapping dimensions of visual streetscape experience rather than mutually exclusive neighborhood categories. Their individual coefficients in the heterogeneous-treatment analysis should therefore be interpreted as conditional associations with treatment-effect heterogeneity, holding the remaining perception dimensions constant.

The correlations also help clarify the interpretation of the spatial patterns presented in Figure 3c. The categories displayed in the maps are derived from continuous perception scores and should not be interpreted as deterministic descriptions of individual neighborhoods. For example, relatively low boringness across much of Manhattan indicates that these locations received comparatively lower boring scores within the NYC perception distribution; it does not imply that Manhattan is categorically "not boring," nor does it establish that the pattern is caused by any single urban-form characteristic such as building height, verticality, or architectural homogeneity.

Similarly, the present correlation analysis does not treat greenery as an additional perception dimension. Vegetation, buildings, roads, sidewalks, sky, vehicles, and other visible elements constitute physical components of the street scene that may contribute differently to several perception dimensions. Consequently, the correlation between two perception measures should not be interpreted as evidence that a specific physical feature, such as greenery, causes their association. Relationships between visual scene characteristics and the perception scores are examined separately through the built-environment and model-interpretability analyses.

1.4 American Community Survey and Smart Location Detailed Tables

The socioeconomic and demographic data on the census block groups were obtained from the American Community Survey 2022 five-year estimates[6]. Additionally, data on auto-oriented intersections per square mile, total activity density, and total road network density at the block group level (CBG), National Walkability Index scor were sourced from the Environmental Protection Agency's (EPA) Smart Location Database. **Table S2** shows the detailed tables and features collected from each dataset.

2 Empirical Strategy and Causal Identification

This section outlines the empirical framework used to estimate the causal effects of bicycle infrastructure on bikeshare ridership. Building on the OD-level data construction described in Supplementary Note 1, we first define treatment assignment based on route exposure to newly installed bicycle lanes within a staggered adoption setting. We

Table S2: Detailed Tables from American Community Survey (ACS) and Smart Location Database (SDL)

Dataset and Code	Feature Name	Table Description
ACS-B02001	White %<br>Black %<br>Asian %	Percentage of individuals in a census block group who list their race as the given category
ACS-B19013	Median Household Income	Median household income ($) (inflation-adjusted dollars)
ACS-B23025	Unemployment Rate %	Percentage of individuals who are unemployed
SLD-NatWalkInd	Walkability Index Score	Components of the built environment that influence the likelihood of walking
SLD-D1b	Population Density	Gross population density (people/acre) on unprotected land
SLD-D3bao	Auto-oriented Intersections /sqm	Intersection density in terms of auto-oriented intersections /sqm
SLD-D1d	Total Activity Density	Gross activity density (employment + HUs) on unprotected land
SLD-D3a	Total Road Network Density	Total street network density on total land

then implement a propensity score matching approach to construct comparable treated and control routes using pre-treatment characteristics at both trip origins and destinations. Using the matched sample, we estimate treatment effects through a Difference-in-Differences framework that distinguishes between protected and painted bike lanes. To assess the validity of the identification strategy, we further conduct event study analyses to examine pre-treatment trends and dynamic effects, and implement placebo tests to evaluate the robustness of the estimated impacts. Together, these approaches provide a comprehensive and internally consistent framework for causal inference in the presence of non-random infrastructure placement.

2.1 Construction of OD Routes and Treatment Assignment

We construct the analytical units of this study at the origin–destination (OD) route level by integrating Citi Bike station locations with New York City's bicycle lane network. Using the street network, shortest-path bicycle routes between all eligible origin and destination station pairs are generated using OSMnx-based routing, resulting in 35,625 OD routes. Each route is then classified based on the type of bicycle infrastructure it intersects, including protected (Class I) lanes, painted (Class II) lanes, or no lane exposure.

Treatment assignment is defined based on whether an OD route intersects newly installed bicycle infrastructure during the study period (2018–2023). Routes that intersect at least one new Class I or Class II bike lane segment within this period are classified as treated, while routes that do not intersect any new installations are classified as controls. For treated routes, the timing of treatment is determined by the earliest installation date of the relevant bike lane segment along the route. This ensures that each treated route has clearly defined pre- and post-treatment periods for causal analysis. To maintain consistency with the staggered Difference-in-Differences framework, we restrict the sample

to routes with sufficient pre- and post-treatment observations. This ensures that ridership trends can be observed prior to infrastructure installation and compared against post-installation changes. Routes that do not satisfy this condition are excluded from the analysis. The final sample consists of 9,233 treated routes and 18,953 control routes.

These route-level treatment assignments are then merged with Citi Bike trip data to construct monthly ridership measures for each OD pair. By combining OD-level travel flows with time-stamped bicycle lane installations, this framework enables a route-based staggered adoption design in which changes in ridership can be directly attributed to the phased rollout of infrastructure across space and time. This approach improves upon station-level designs by accounting for the relational structure of cycling demand, where travel behavior depends jointly on both origins and destinations.

2.2 Propensity Score Matching

Before estimating treatment effects using the staggered Difference-in-Differences framework, we refine the selection of control routes using propensity score matching (PSM). Unlike conventional station-level implementations, our matching procedure is conducted at the OD-route level, ensuring that treated routes are compared with observationally similar control routes that share comparable origin and destination characteristics as well as pre-treatment demand patterns. Treated routes are defined as those exposed to newly installed protected or painted bicycle lanes, while control routes consist of untreated routes observed within the same installation year cohort. This staggered matching design improves causal identification by jointly accounting for treatment timing and the spatial context of both trip endpoints.

To estimate the probability of treatment assignment, we fit a logistic regression model in which the treatment indicator is regressed on pre-treatment route characteristics:

$$\Pr(T_i = 1 \mid X_i) = \frac{\exp(\eta_i)}{1 + \exp(\eta_i)} \tag{2}$$

where

$$\eta_i = \beta_0 + \beta_1 \ln(\text{BaselineTrips}_i) + \gamma_{y(i)} + \boldsymbol{\theta}^\top \mathbf{O}_i + \boldsymbol{\delta}^\top \mathbf{D}_i \tag{3}$$

Here, $T_i = 1$ indicates that route i is assigned to treatment, $\ln(\text{BaselineTrips}_i)$ represents pre-treatment ridership, and $\gamma_{y(i)}$ captures installation-year fixed effects that align routes within the same staggered rollout cohort. The vectors $\mathbf{O}_i$ and $\mathbf{D}_i$ denote origin- and destination-level covariates, respectively. This model has an accuracy score of 0.836, demonstrating reasonable predictive performance. Detailed regression results are presented in Supplementary **Table S3**.

The covariates are selected to capture factors associated with both bicycle lane placement and cycling demand. These include built-environment characteristics from the Environmental Protection Agency's Smart Location Database, such as walkability, population density, road density, intersection density, activity density, and network accessibility [7]. We also incorporate sociodemographic attributes from the American Community Survey 2023 five-year estimates, including median household income, racial composition, and unemployment rates [6]. In addition, baseline route-level demand is measured using pre-treatment Citi Bike ridership, and machine learning-derived streetscape perception indicators, including beautiful, boring, depressing, livable, safe, and wealthy, are incorporated for both origins and destinations.

We implement one-to-one nearest-neighbor matching without replacement. To preserve temporal consistency under staggered treatment adoption, treated routes are matched only to control routes within the same installation year. Within each year-specific stratum, matches are selected based on the nearest propensity score subject to a caliper of 0.02, ensuring that only sufficiently similar treated and control routes are paired. This procedure yields a matched sample of 5,466 treated routes and 5,466 control routes, resulting in 10,932 route observations for subsequent analysis. **Table S4** provide descriptive statistics for the matched data obtained using staggered matching approach.

Following matching, we evaluate covariate balance using standardized mean differences (SMDs) and propensity score overlap diagnostics. The SMD for covariate k is defined as:

$$\text{SMD}_k = \frac{\bar{x}_{k,T} - \bar{x}_{k,C}}{\sqrt{\frac{s^2_{k,T} + s^2_{k,C}}{2}}} \tag{4}$$

where $\bar{x}_{k,T}$ and $\bar{x}_{k,C}$ denote the matched sample means for treated and control routes, and $s^2_{k,T}$ and $s^2_{k,C}$ are the corresponding variances. An SMD below 0.10 indicates sufficient overlap between groups and suggests that the matching procedure has effectively reduced observable imbalance. In addition, the convergence of propensity score distributions between treated and control routes provides further evidence of improved comparability.

Overall, this matching stage is critical to the identification strategy. By pairing treated routes with untreated routes that share similar pre-treatment ridership, comparable origin and destination contexts, and aligned treatment timing, the subsequent Difference-in-Differences and heterogeneous treatment effect analyses rely on a substantially more credible counterfactual comparison.

Table S3: Logistic Regression Predicting Treatment Assignment

**Variable**	**Coefficient**
Intercept	-4.3502 (3.573)
Installation year fixed effects	
2019	0.0982 (0.062)
2020	-0.1121^{*} (0.060)
2021	-0.4648^{***} (0.059)
2022	-0.3905^{***} (0.054)
2023	-0.3446^{***} (0.053)
**Baseline demand**	
log(Baseline Pre-Trips)	-0.1531^{***} (0.011)
**Origin characteristics**	
Median Household Income	4.471×10^{-7} (3.23×10^{-7})
White Population	9.472×10^{-5} *** (3.55×10^{-5})
Black Population	-0.0004^{***} (4.69×10^{-5})
Asian Population	0.0002^{***} (5.92×10^{-5})
Unemployment Rate	0.0204^{***} (0.001)
Walkability	0.0076 (0.006)
Population Density	-0.1646^{***} (0.018)

Continued on next page

Variable	Coefficient
Road Density	0.0368 (0.025)
Intersection Density	-0.0495^{***} (0.016)
Activity Density	0.0271 (0.018)
Beautiful	-1.2209 (1.399)
Boring	-6.6821^{***} (1.621)
Depressing	10.0274^{***} (3.022)
Lively	-2.7175^{***} (0.898)
Safe	-1.1680 (1.361)
Wealthy	11.2121^{***} (2.124)
**Destination characteristics**	
Median Household Income	-3.798×10^{-7} (3.28×10^{-7})
White Population	$9.472 \times 10^{-5***}$ (3.55×10^{-5})
Black Population	-0.0004^{***} (4.60×10^{-5})
Asian Population	0.0004^{***} (6.10×10^{-5})
Unemployment Rate	0.0202^{***} (0.001)
Walkability	0.0119^{*} (0.007)
Population Density	-0.0989^{***} (0.018)
Road Density	0.0889^{***} (0.026)
Intersection Density	-0.0629^{***} (0.016)
Activity Density	0.0841^{***} (0.017)
Road Network Density	-0.1366^{***} (0.050)
Beautiful	0.1089 (1.418)
Boring	-8.5272^{***} (1.691)
Depressing	6.8191^{**} (3.031)
Lively	-4.7617^{***} (0.942)
Safe	0.9391 (1.426)
Wealthy	4.2786^{**} (2.111)
**Model statistics**	
Observations	28,186
Pseudo R^2	0.0526
Log-Likelihood	$-17,179$
LL-Null	$-18,132$
Likelihood Ratio p-value	< 0.001

Notes: Standard errors in parentheses. $^{***}p < 0.01$, $^{**}p < 0.05$, $^{*}p < 0.1$.

Table S4: Descriptive Statistics for Bike Lane Treated and Matched Control OD Routes

Variable	N	Mean	Std. dev	Min	Max
**Treated group**					
Baseline travel variable					
Log baseline pre-trips	5466	4.132	1.562	0.693	8.358
Origin covariates					
Origin median household income	5466	112021.675	65124.092	11406	250001

Continued on next page

Variable	N	Mean	Std. dev	Min	Max
Origin white population	5466	597.982	527.836	0	6227
Origin black population	5466	222.048	332.238	0	2812
Origin Asian population	5466	171.844	263.416	0	2469
Origin unemployment rate	5466	8.395	10.410	0	100
Origin walkability index	5466	14.649	2.475	6.000	19.833
Origin population density	5466	4.188	1.154	0	6.036
Origin road density	5466	0.518	0.991	0	4.041
Origin intersection density	5466	0.664	1.490	0	6.029
Origin activity density	5466	4.409	1.313	0	7.799
Origin road network	5466	3.477	0.389	0	4.685
Origin beautiful score	5466	0.385	0.075	0.219	0.780
Origin boring score	5466	0.435	0.046	0.350	0.608
Origin depressing score	5466	0.518	0.048	0.297	0.660
Origin lively score	5466	0.607	0.078	0.338	0.762
Origin safety score	5466	0.409	0.073	0.255	0.717
Origin wealthy score	5466	0.457	0.047	0.323	0.675
Destination covariates					
Destination median household income	5466	111802.075	64449.415	11406	250001
Destination white population	5466	609.448	539.835	0	6227
Destination black population	5466	222.171	328.592	0	2812
Destination Asian population	5466	175.325	267.935	0	2469
Destination unemployment rate	5466	8.446	10.520	0	100
Destination walkability index	5466	14.601	2.470	6.000	19.833
Destination population density	5466	4.196	1.169	0	6.643
Destination road density	5466	0.526	0.990	0	4.041
Destination intersection density	5466	0.665	1.494	0	6.029
Destination activity density	5466	4.413	1.307	0	7.799
Destination road network	5466	3.481	0.351	0	4.685
Destination beautiful score	5466	0.386	0.072	0.214	0.780
Destination boring score	5466	0.435	0.045	0.350	0.611
Destination depressing score	5466	0.518	0.046	0.297	0.669
Destination lively score	5466	0.607	0.077	0.338	0.762
Destination safety score	5466	0.410	0.071	0.248	0.717
Destination wealthy score	5466	0.457	0.045	0.316	0.675
**Matched control group**					
Baseline travel variable					
Log baseline pre-trips	5466	4.146	1.514	0.693	8.502
Origin covariates					
Origin median household income	5466	113829.020	61235.040	11406	250001
Origin white population	5466	599.964	518.411	0	6227
Origin black population	5466	211.713	335.627	0	2709
Origin Asian population	5466	165.864	269.552	0	2469
Origin unemployment rate	5466	8.353	10.896	0	100
Origin walkability index	5466	14.654	2.552	6.000	19.833

Continued on next page

Variable	N	Mean	Std. dev	Min	Max
Origin population density	5466	4.209	1.006	0	6.643
Origin road density	5466	0.529	1.000	0	4.376
Origin intersection density	5466	0.704	1.546	0	6.029
Origin activity density	5466	4.429	1.193	0	7.799
Origin road network	5466	3.493	0.318	0	5.035
Origin beautiful score	5466	0.385	0.068	0.214	0.729
Origin boring score	5466	0.434	0.045	0.346	0.611
Origin depressing score	5466	0.518	0.046	0.321	0.669
Origin lively score	5466	0.608	0.079	0.331	0.770
Origin safety score	5466	0.409	0.069	0.248	0.694
Origin wealthy score	5466	0.457	0.047	0.316	0.653
Destination covariates					
Destination median household income	5466	112149.741	60128.169	11406	250001
Destination white population	5466	608.893	502.811	0	4427
Destination black population	5466	216.863	340.030	0	2812
Destination Asian population	5466	168.999	260.071	0	2469
Destination unemployment rate	5466	8.307	10.466	0	100
Destination walkability index	5466	14.585	2.493	6.000	19.833
Destination population density	5466	4.186	1.014	0	6.643
Destination road density	5466	0.544	1.007	0	4.041
Destination intersection density	5466	0.686	1.536	0	6.029
Destination activity density	5466	4.432	1.226	0	7.799
Destination road network	5466	3.488	0.343	0	4.685
Destination beautiful score	5466	0.387	0.069	0.214	0.780
Destination boring score	5466	0.435	0.046	0.346	0.611
Destination depressing score	5466	0.518	0.047	0.297	0.669
Destination lively score	5466	0.607	0.080	0.331	0.770
Destination safety score	5466	0.410	0.070	0.248	0.717
Destination wealthy score	5466	0.457	0.048	0.316	0.675

2.3 Difference-in-differences Analysis Across Bike Lane Type

Following the construction of matched treated and control route pairs using the PSM framework, we estimate the causal effect of bicycle infrastructure on ridership using a Difference-in-Differences (DiD) specification that distinguishes between painted and protected bike lanes. Because bicycle lanes were introduced at different times across routes, this framework exploits staggered treatment timing while controlling for both route-specific and common temporal confounders.

Treatment is defined at the route-month level. The treatment indicator W_{it} is equal to 1 for treated routes once the installation month and year of the relevant bicycle lane segment has been reached, and 0 otherwise. For control routes, W_{it} remains zero throughout the study period. This setup allows for a comparison of pre- and post-treatment changes in ridership between treated and matched control routes.

The DID specification is defined as:

$$\ln(Y_{it}) = \alpha + \beta_0 W_{it}\mathbf{1}(C_i = 0) + \beta_1 W_{it}\mathbf{1}(C_i = 1) + \mu_i + \lambda_t + \varepsilon_{it} \quad (5)$$

where Y_{it} represents the 'number of bike trips +1' observed on OD route i in month t, allowing the logarithmic transformation to accommodate zero-trip observations. This semi-log specification reduces right-skewness in ridership counts while preserving observations with no recorded trips. The variable $C_i \in \{0, 1\}$ denotes the type of bicycle lane treatment assigned to route i, where $C_i = 0$ indicates a painted bike lane and $C_i = 1$ indicates a protected bike lane.

The coefficients β_0 and β_1 capture the average treatment effects of painted and protected bike lanes, respectively. By allowing separate coefficients for each facility type, the model explicitly accounts for heterogeneous treatment effects across infrastructure designs. The term μ_i represents route-level fixed effects, which absorb all time-invariant differences across OD routes. The term λ_t denotes time fixed effects (year and month). The error term ε_{it} captures idiosyncratic variation. This specification enables a direct comparison of whether low-stress protected bicycle lanes generate larger ridership gains than conventional painted infrastructure within a unified causal framework. Supplementary **Table S5** shows the full regression results.

Table S5: Effect of Treatment on Bike Trips for both Protected and Painted Bike Lanes

Constant	4.5947***
	(0.019), $p < 0.001$
Treated × Post × Painted Bike Lane	0.0967**
	(0.035), $p = 0.005$
Treated × Post × Protected Bike Lane	0.2705***
	(0.029), $p < 0.001$
Route Fixed Effects	Yes
Year-Month Fixed Effects	Yes
Observations	21,864
R^2	0.902
Adjusted R^2	0.803

Notes: Standard errors in parentheses. Robust standard errors are clustered at the route level. $^{***}p < 0.01$, $^{**}p < 0.05$, $^{*}p < 0.1$.

2.4 Destination-specific route information and alternative-route sensitivity

The primary analysis represents Citi Bike travel demand at the origin–destination (OD) level rather than aggregating all trips around individual stations. This representation allows trips departing from the same origin station to retain different infrastructure exposures depending on their destinations. Because the observed Citi Bike records provide trip origins and destinations but not GPS trajectories, the main analysis represents each OD pair using an inferred network path. We therefore conducted two additional diagnostics to assess: (1) how much destination-specific infrastructure information is retained by the OD-pair representation relative to origin-station aggregation; and (2) whether route-level infrastructure exposure is sensitive to replacing the single-shortest-path representation with a set of plausible alternative routes.

2.4.1 Destination-specific information retained by the OD-pair representation

We first examined whether trips departing from the same Citi Bike origin station encounter meaningfully different bicycle-infrastructure environments depending on their destinations. A fixed random sample of 5,000 OD pairs was selected from the study data, of which 4,854 could be successfully routed through the street network.

For each OD route, we calculated two infrastructure-exposure measures. Overall bicycle-infrastructure exposure represents the proportion of route length containing bicycle infrastructure, while protected-lane exposure represents the proportion of route length containing protected bicycle infrastructure. We then grouped routes by their origin station and retained origins represented by at least two sampled destination-specific OD pairs. This resulted in 942 origin stations.

For infrastructure measure m, the within-origin exposure range was calculated as

$$R_o^{(m)} = \max_{d \in D_o} E_{od}^{(m)} - \min_{d \in D_o} E_{od}^{(m)}, \tag{6}$$

where $E_{od}^{(m)}$ denotes infrastructure exposure for the route connecting origin o to destination d, and D_o denotes the set of sampled destinations associated with origin o. A larger value therefore indicates greater variation in infrastructure exposure among trips departing from the same station.

Among the 942 origin stations represented by at least two sampled OD pairs, 69.4% were connected to routes spanning at least two different dominant bicycle-infrastructure classes (**Table S6**). The median within-origin range in overall bicycle-infrastructure exposure was 0.480, indicating that the least and most infrastructure-exposed routes from the median origin differed by 48.0 percentage points of route length. The corresponding median range in protected-lane exposure was 0.336, corresponding to a 33.6-percentage-point difference.

Figure 5 shows the complete distribution of within-origin variation in protected-lane exposure. Although some origin stations exhibit little variation among destination routes, the distribution extends across a wide range of values, with a median of 0.336. This indicates that trips sharing an origin station can encounter substantially different protected-lane environments depending on their destinations.

We further examined spatial overlap among routes sharing the same origin using the Jaccard index. The median pairwise route overlap was 0.008, indicating that routes connecting the same origin to different destinations generally diverge and traverse different parts of the street network. Because these routes terminate at different destinations, this measure is interpreted as an indicator of destination-specific corridor divergence rather than route-prediction accuracy.

Finally, we examined whether infrastructure exposure occurring farther along an OD route could be missed by a representation focusing only on the immediate vicinity of the origin. Among trips with no bicycle infrastructure encountered within the first 100 m of the inferred route, the trip-weighted probability of encountering bicycle infrastructure farther along the route was 39.0%. Here, the 100-m threshold refers to network distance along the inferred route rather than a Euclidean station buffer.

Together, these results show that an origin station cannot necessarily be characterized by a single bicycle-infrastructure environment. Retaining the OD-pair unit therefore preserves destination-specific information about the infrastructure encountered between

trip origins and destinations that would otherwise be collapsed under origin-station aggregation.

Table S6: Destination-specific information retained by the OD-pair representation.

Diagnostic	Result
Sampled OD pairs	5,000
Successfully routed OD pairs	4,854
Origin stations represented by ≥ 2 sampled OD pairs	942
Origins spanning ≥ 2 dominant infrastructure classes	69.4%
Median within-origin range: overall bicycle infrastructure	0.480
Median within-origin range: protected-lane exposure	0.336
Median within-origin pairwise route overlap (Jaccard)	0.008
Trip-weighted probability of infrastructure beyond first 100 m	39.0%

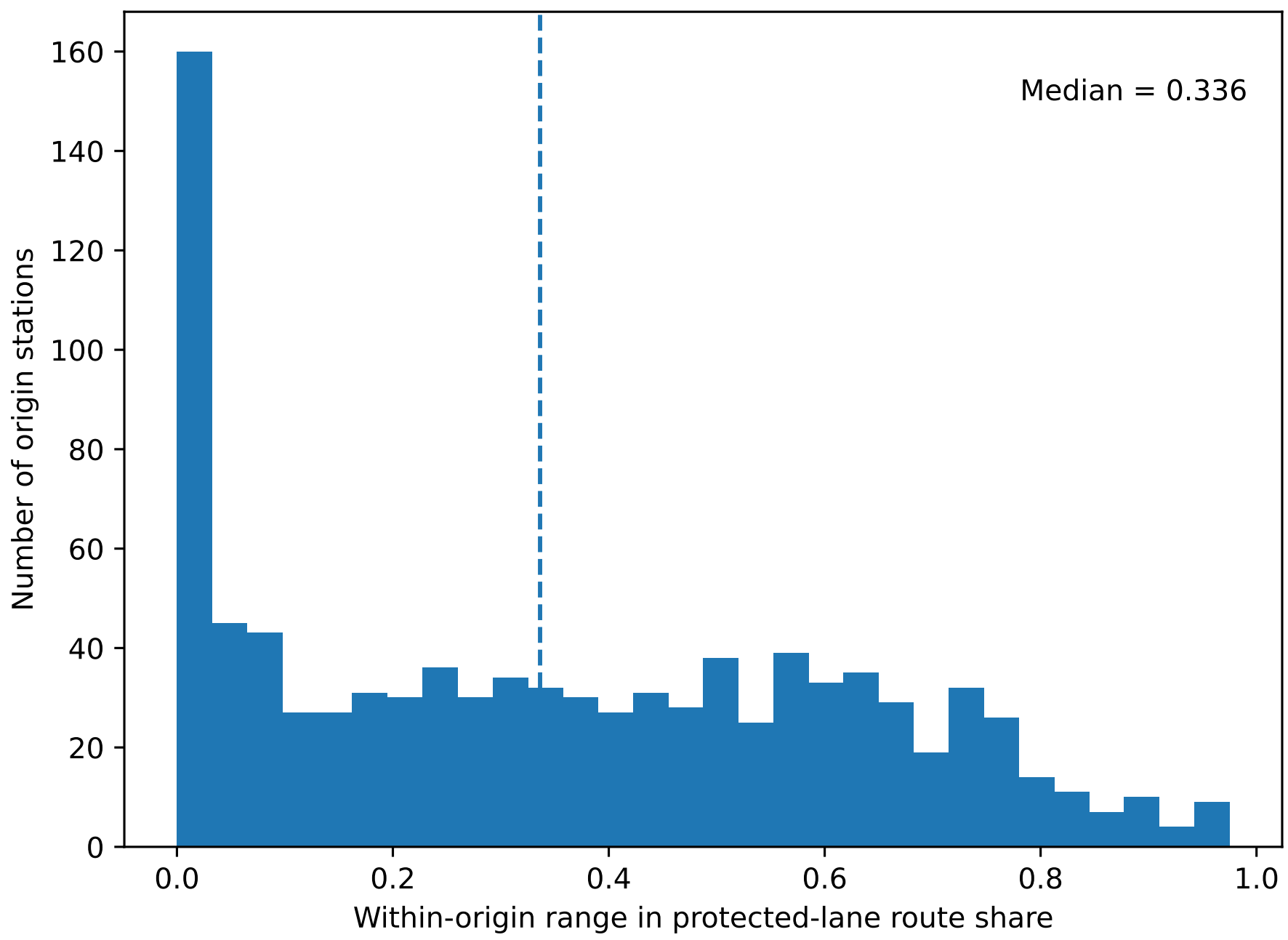

Figure S5: **Destination-specific variation in protected-lane exposure among routes sharing the same origin station.** Distribution of the within-origin range in protected-lane route exposure across origin stations represented by at least two sampled OD pairs. The range is calculated as the difference between the maximum and minimum protected-lane exposure among destination-specific routes sharing the same origin. The median within-origin range is 0.336, indicating substantial destination-specific variation in protected-lane environments encountered by trips departing from the same Citi Bike station.

2.4.2 Sensitivity to plausible alternative-route assignments

Because the public Citi Bike trip records do not contain observed GPS trajectories, the primary route representation assumes an inferred shortest network path between each origin and destination. To evaluate whether the resulting infrastructure-exposure

measures are strongly dependent on this assumption, we conducted an alternative-route sensitivity analysis using the same fixed random sample of 5,000 OD pairs.

For each OD pair, we generated up to five plausible network routes and retained alternatives whose route length did not exceed 1.5 times the shortest-route length. Rather than assigning each trip exclusively to one route, probabilities were distributed across the retained alternatives using a Path-Size Logit (PSL) formulation. This approach accounts for both route length and the degree of overlap among alternative paths.

For OD pair i, the probability of selecting alternative route r was represented as

$$P_{ir} = \frac{\exp(V_{ir})}{\sum_{q \in C_i} \exp(V_{iq})}, \tag{7}$$

where C_i denotes the set of retained alternative routes and V_{ir} denotes the route utility incorporating route length and a path-size correction for overlap among alternatives.

Probability-weighted infrastructure exposure was then calculated as

$$\bar{E}_i^{(m)} = \sum_{r \in C_i} P_{ir} E_{ir}^{(m)}, \tag{8}$$

where $E_{ir}^{(m)}$ denotes exposure to infrastructure type m along alternative route r. We compared these probability-weighted measures with the corresponding exposure obtained from the deterministic shortest-path representation.

Among the 4,854 successfully routed OD pairs, 98.3% had more than one plausible route, and the median number of retained candidate routes was five (**Table S7**). The median probability assigned to the shortest route was 0.226, indicating that probability was generally distributed across several plausible alternatives rather than concentrated entirely on the shortest route.

Despite this route-choice variation, infrastructure-exposure measures were highly consistent across the deterministic and stochastic representations. For protected-lane exposure, the comparison yielded Pearson $r = 0.977$, Spearman $\rho = 0.969$, and a mean absolute error (MAE) of 0.028. Thus, the average absolute difference between the two protected-lane exposure measures was approximately 2.8 percentage points of route length.

For overall bicycle-infrastructure exposure, the corresponding values were Pearson $r = 0.963$, Spearman $\rho = 0.964$, and MAE $= 0.048$, corresponding to an average absolute difference of approximately 4.8 percentage points. The dominant infrastructure classification was also unchanged between the deterministic and stochastic representations for 93.1% of OD pairs.

Figure S6 further illustrates this agreement for protected-lane exposure. Most observations lie close to the one-to-one line, consistent with the high rank correlation ($\rho = 0.969$) and low MAE (0.028). This indicates that allowing multiple plausible routes introduces route-choice uncertainty without substantially changing the protected-lane exposure assigned to most OD pairs.

We additionally evaluated the similarity and length of the probability-weighted alternative routes. The trip-weighted expected Jaccard overlap with the shortest route was 0.766, indicating that the plausible alternatives generally shared a substantial proportion of their network segments with the deterministic route. The median expected route-length ratio relative to the shortest path was 1.003, suggesting that probability was concentrated primarily on alternatives with similar travel distances.

These findings indicate that although multiple plausible network routes exist for most OD pairs, the resulting bicycle-infrastructure exposure measures are comparatively stable to alternative-route assignment. Importantly, this analysis evaluates the sensitivity of infrastructure-exposure measurement rather than validating the inferred routes against cyclists' actual trajectories. Observed GPS or app-based trajectory data would still provide a more direct measure of experienced route choice and infrastructure exposure.

Table S7: Sensitivity of infrastructure exposure to alternative-route assignment.

**Diagnostic**	**Result**
Successfully routed OD pairs	4,854
OD pairs with > 1 plausible route	98.3%
Median number of retained candidate routes	5.0
Median probability assigned to shortest route	0.226
Trip-weighted expected Jaccard overlap with shortest route	0.766
Median expected route-length ratio	1.003
Protected-lane exposure: Pearson r	0.977
Protected-lane exposure: Spearman ρ	0.969
Protected-lane exposure: MAE	0.028
Overall bicycle-infrastructure exposure: Pearson r	0.963
Overall bicycle-infrastructure exposure: Spearman ρ	0.964
Overall bicycle-infrastructure exposure: MAE	0.048
Dominant infrastructure-class agreement	93.1%

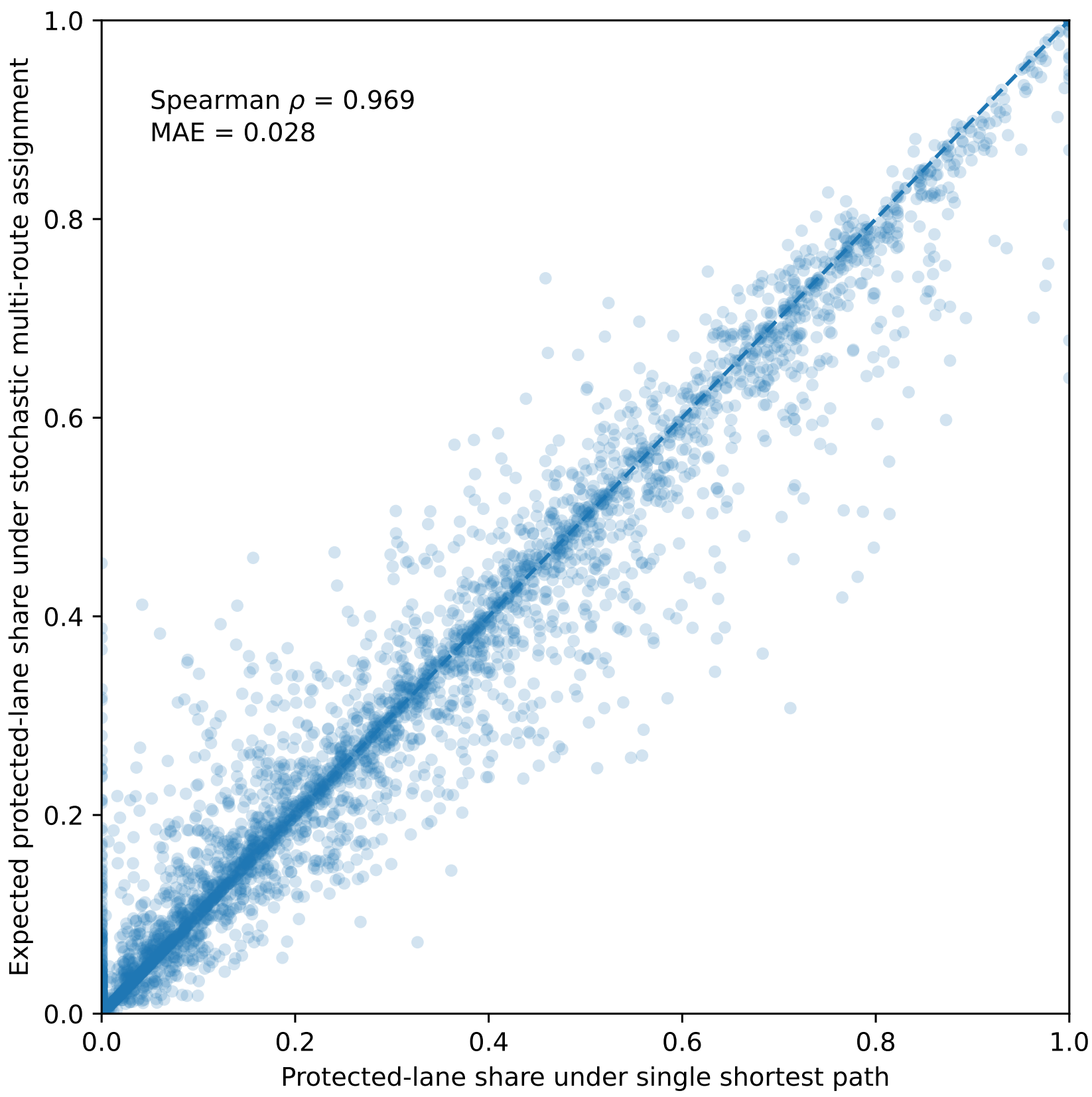

Figure S6: Sensitivity of protected-lane exposure to alternative-route assignment.

3 Heterogeneous Treatment Effects and Perception-Based Mechanisms

This section investigates how the causal impacts of bicycle infrastructure vary across different urban contexts and perceived streetscape conditions. Building on the average treatment effects estimated using the staggered Difference-in-Differences framework, we examine treatment heterogeneity using a multi-arm causal forest model that captures nonlinear and context-dependent responses to painted and protected bike lanes (**Section 3.1**). We then explore how perceived neighborhood environments shape these effects through perception-based moderation analyses, first using univariate specifications to isolate the influence of individual perception dimensions, and then extending to multivariate models that jointly account for multiple perceptual attributes (**Section 3.2**).

To translate these relationships into interpretable outcomes, we compute predicted treatment effects across the observed distribution of perception scores, illustrating how infrastructure performance varies continuously with perceived streetscape quality (**Section 3.3**). We further decompose these predicted effects to isolate the marginal contribution of perception variables independent of baseline infrastructure effects, providing additional insight into the mechanisms driving treatment heterogeneity (**Section 3.4**). Finally, we apply the estimated models to generate street-level scenario predictions for untreated candidate corridors, demonstrating how heterogeneous treatment estimates can inform context-sensitive infrastructure planning and policy decisions (**Section 3.5**).

3.1 Heterogeneous Treatment Effects Using a Multi-Arm Causal Forest

To examine whether the effectiveness of bicycle infrastructure varies across route contexts, we estimate heterogeneous treatment effects using a multi-arm causal forest model. We construct the causal forest sample using the propensity-score-matched treated and control routes described in Supplementary Note 2.2. For each route i, the outcome variable is defined as the change in logged ridership between the post- and pre-treatment periods:

$$\Delta \ln(Y_i) = \ln(\text{PostTrips}_i) - \ln(\text{PreTrips}_i) \tag{9}$$

which captures proportional changes in cycling activity while accommodating zero-trip observations. Treatment assignment is specified as a multi-valued variable $W_i \in \{0, 1, 2\}$, where $W_i = 0$ denotes control routes, $W_i = 1$ denotes routes exposed to painted bike lanes, and $W_i = 2$ denotes routes exposed to protected bike lanes.

The causal forest estimates the conditional average treatment effect (CATE):

$$\tau_k(x) = \mathbb{E}\left[Y_i(k) - Y_i(0) \mid X_i = x\right], \quad k \in \{1, 2\}. \tag{10}$$

where X_i includes pre-treatment route characteristics such as baseline ridership, origin- and destination-level built environment attributes, socio-economic indicators, and machine learning-derived perception measures.

Following the double machine learning framework, the `CausalForestDML` estimator first models the outcome and treatment assignment processes using random forests and then estimates treatment effects using orthogonalized residuals. This approach improves robustness to model misspecification and enables flexible, nonlinear heterogeneity without requiring pre-specified interaction terms. The resulting average treatment effects from the

causal forest align closely with the DiD estimates, providing an important validation of the main causal findings. Specifically, the estimated average treatment effect for painted bike lanes is 0.077 (95% CI: $[0.003,\ 0.151]$, $p = 0.041$), indicating a small and statistically significant effect. In contrast, protected bike lanes yield a larger and statistically significant effect of 0.276 (95% CI: $[0.036, 0.517]$, $p = 0.024$). These estimates are consistent with the DiD results, which similarly indicate that protected lanes generate substantially larger ridership gains than painted lanes. This consistency across quasi-experimental and machine learning approaches strengthens confidence in the robustness of the estimated treatment effects.

3.2 Perception based Moderation of Treatment Effects

To identify which perceived neighborhood characteristics explain the heterogeneity detected by the causal forest, we examine how route-level treatment effects vary with perceived streetscape attributes. We begin with univariate moderation analyses to isolate the influence of each perception dimension independently, and then extend to multivariate models that jointly account for correlations across perceptions.

3.2.1 Univariate Moderation Analysis

We first estimate separate linear models for each perception variable, relating route-level treatment effects to standardized perception scores. This approach provides a direct interpretation of how each perceived attribute individually correlates with infrastructure effectiveness. Across both lane types, several consistent patterns emerge. Perceived safety exhibits a strong positive association with treatment effects for both painted ($\beta = 0.470,\ p < 0.001$) and protected lanes ($\beta = 0.416,\ p < 0.001$), suggesting that infrastructure performs better in environments already perceived as safe. Similarly, perceived beauty is positively associated with treatment effects, with stronger effects for protected lanes ($\beta = 0.243,\ p < 0.001$) than painted lanes ($\beta = 0.150,\ p < 0.001$), indicating that visually attractive streetscapes amplify the benefits of higher-quality infrastructure. In contrast, perceived livability shows a negative relationship with treatment effects for both painted ($\beta = -0.312,\ p < 0.001$) and protected lanes ($\beta = -0.309,\ p < 0.001$), suggesting that infrastructure generates larger marginal gains in less livable environments, consistent with a compensatory mechanism. Perceived boringness exhibits the strongest positive association among all variables, with large coefficients for both painted ($\beta = 1.485,\ p < 0.001$) and protected lanes ($\beta = 1.276,\ p < 0.001$), indicating that infrastructure may yield substantial gains in visually monotonous settings. Perceived wealth and depressingness show weaker and less consistent relationships. Wealth is statistically insignificant for painted lanes and only marginally significant for protected lanes, while depressingness is not statistically significant for either lane type. These patterns suggest that not all perception dimensions equally contribute to treatment heterogeneity when considered in isolation. Supplementary **Figure S7** presents the forest plot of univariate moderation effects, while Supplementary **Table S8** reports the corresponding regression results.

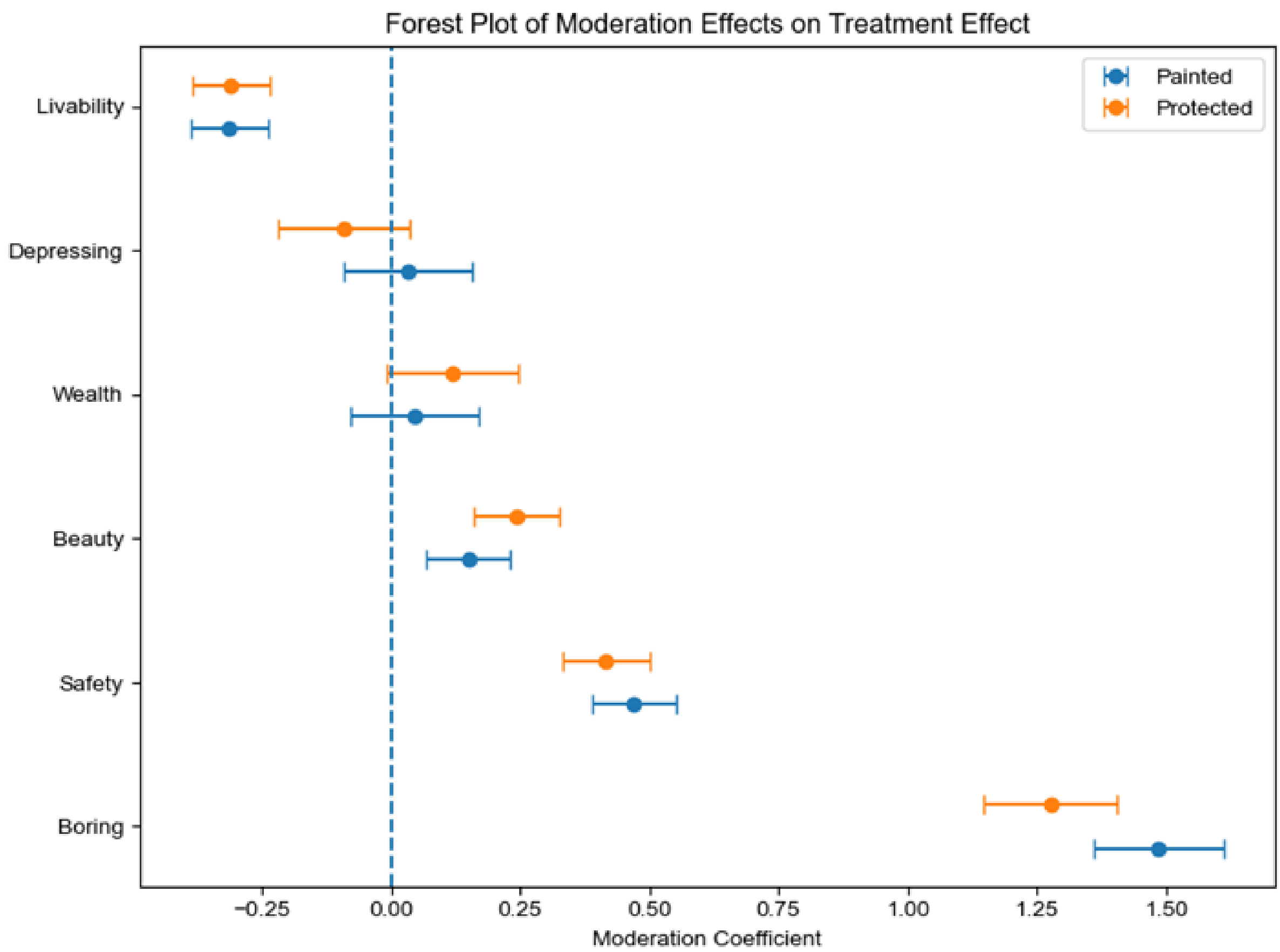

Figure S7: Forest Plot of Moderation Effeccts on Treatment Effects

Table S8: Univariate Perception based Moderation of Route Level Treatment Effects

**Perception**	**Lane type**	**Coefficient**	**95% CI**	***p*-value**
**Safe**	Painted	0.4697^{***}	$[0.388,\ 0.551]$	< 0.001
	Protected	0.4159^{***}	$[0.332,\ 0.500]$	< 0.001
**Livable**	Painted	-0.3123^{***}	$[-0.386,\ -0.239]$	< 0.001
	Protected	-0.3093^{***}	$[-0.385,\ -0.233]$	< 0.001
**Beautiful**	Painted	0.1503^{***}	$[0.069,\ 0.232]$	< 0.001
	Protected	0.2426^{***}	$[0.159,\ 0.326]$	< 0.001
**Wealthy**	Painted	0.0456	$[-0.078,\ 0.169]$	0.470
	Protected	0.1192^{*}	$[-0.008,\ 0.247]$	0.067
**Depressing**	Painted	0.0320	$[-0.092,\ 0.156]$	0.613
	Protected	-0.0912	$[-0.219,\ 0.037]$	0.162
**Boring**	Painted	1.4854^{***}	$[1.361,\ 1.610]$	< 0.001
	Protected	1.2761^{***}	$[1.147,\ 1.405]$	< 0.001

Note.*** $p < 0.001$; ** $p < 0.01$; * $p < 0.10$.

3.2.2 Multivariate Moderation Analysis

To account for correlations across perception variables, we next estimate multivariate moderation models in which all perception dimensions are included simultaneously. For each lane type $l \in \{\text{painted}, \text{protected}\}$, we estimate:

$$\tau_i^{(l)} = \alpha^{(l)} + \sum_{k=1}^{6} \beta_k^{(l)} z_{ik} + \varepsilon_i \tag{11}$$

where $\tau_i^{(l)}$ denotes the route-level treatment effect estimated from the causal forest, and z_{ik} represents standardized perception scores. All models are estimated using ordinary least squares with heteroskedasticity-robust (HC3) standard errors. The multivariate results reveal important shifts relative to the univariate analysis. For painted lanes, perceived liveliness ($\beta = 0.200,\ p < 0.001$) and boringness ($\beta = 0.234,\ p < 0.001$) remain strong positive predictors of treatment effects, while safety becomes more moderate but remains significant ($\beta = 0.067,\ p < 0.001$). In contrast, beauty and wealth become statistically insignificant once other perceptions are controlled for, suggesting that their univariate effects were partly driven by correlation with other dimensions. Perceived depressingness remains positive and significant ($\beta = 0.081,\ p = 0.002$). For protected lanes, several relationships differ notably. Perceived beauty becomes a strong and significant positive predictor ($\beta = 0.157,\ p < 0.001$), while perceived safety reverses sign and becomes negatively associated with treatment effects ($\beta = -0.098,\ p < 0.001$). This suggests that protected lanes generate larger marginal gains in environments perceived as less safe, consistent with their role in reducing traffic-related stress. As with painted lanes, boringness ($\beta = 0.276,\ p < 0.001$) and liveliness ($\beta = 0.255,\ p < 0.001$) remain strong positive predictors. Wealth and depressingness are not statistically significant in the multivariate specification. The intercept terms further highlight baseline differences across lane types. The estimated baseline effect is substantially larger for protected lanes ($\alpha = 0.317,\ p < 0.001$) than for painted lanes ($\alpha = 0.042,\ p < 0.001$), indicating that even in an average perceived environment, protected infrastructure generates markedly higher ridership gains (see **Figure 7**). Taken together, the comparison between univariate and multivariate models shows that some perception effects are robust (e.g., boringness and liveliness), while others depend on the broader perceptual context (e.g., safety and beauty). These results highlight the importance of accounting for correlated perception dimensions when interpreting heterogeneous treatment effects. Supplementary Table **Table S9** shows the full regression results.

Table S9: Multivariate Perception based Moderation of Route Level Treatment Effects

	**Painted lanes**	**Protected lanes**
Constant	0.0420*** (0.003)	0.3172*** (0.003)
Perceived safety	0.0674*** (0.018)	−0.0982*** (0.021)
Perceived beauty	0.0270 (0.018)	0.1567*** (0.020)
Perceived livability	0.2003*** (0.014)	0.2547*** (0.016)
Perceived wealth	−0.0101 (0.017)	−0.0317* (0.019)
Perceived boringness	0.2342*** (0.013)	0.2760*** (0.015)
Perceived depressingness	0.0805*** (0.026)	0.0298 (0.030)
Observations	10,932	10,932
R^2	0.141	0.092
Adj. R^2	0.140	0.091

Notes: Standard errors in parentheses. Heteroskedasticity-robust (HC3) standard errors are used.
$^{***}p < 0.01$, $^{**}p < 0.05$, $^{*}p < 0.1$.

3.3 Spatial autocorrelation of perception-conditioned treatment-effect patterns

To formally assess the spatial structure shown in Figure 3c, we conducted global and local spatial-autocorrelation analyses for each of the six perception dimensions: beautiful, livable, safe, wealthy, boring, and depressing. Figure 3c maps the perception-conditioned relative advantage of protected over painted bicycle lanes across New York City Census Block Groups (CBGs). For perception dimension k, this relative advantage is defined as

$$\Delta_k(z_k) = \hat{\tau}_k^{(R)}(z_k) - \hat{\tau}_k^{(P)}(z_k) = \left(\hat{\beta}_k^{(R)} - \hat{\beta}_k^{(P)}\right) z_k, \tag{12}$$

where z_k denotes the standardized perception score, and $\hat{\beta}_k^{(R)}$ and $\hat{\beta}_k^{(P)}$ denote the estimated moderation coefficients for protected and painted bicycle lanes, respectively. Positive values of $\Delta_k(z_k)$ indicate a greater model-implied relative advantage for protected lanes, whereas negative values indicate a relative advantage for painted lanes.

Because $\Delta_k(z_k)$ is a linear transformation of the standardized perception score for each dimension, the spatial-autocorrelation analysis evaluates the geographic organization of the model-implied perception-conditioned relative advantage surface. It should therefore not be interpreted as an independent spatial test of observed treatment effects or cycling responses.

3.3.1 Global spatial autocorrelation

Spatial relationships between CBGs were defined using first-order Queen contiguity, under which two CBGs are considered neighbors when they share either a boundary or a vertex. The resulting spatial-weight matrix was row-standardized.

For each perception-conditioned surface, we calculated Global Moran's I:

$$I = \frac{N}{S_0} \frac{\sum_i \sum_j w_{ij}(x_i - \bar{x})(x_j - \bar{x})}{\sum_i (x_i - \bar{x})^2}, \tag{13}$$

where N denotes the number of spatial units, x_i is the perception-conditioned relative advantage for CBG i, $\bar{x}$ is its spatial mean, w_{ij} is the spatial weight between CBGs i and j, and $S_0 = \sum_i \sum_j w_{ij}$. Statistical significance was evaluated using 9,999 random permutations of the observed values across the spatial units.

All six dimensions exhibited significant positive spatial autocorrelation (**Table S10**). Global Moran's I ranged from 0.472 for wealthy to 0.710 for boring, with permutation-based $p < 0.001$ in every case. The strongest spatial autocorrelation was observed for boring ($I = 0.710$) and livable ($I = 0.665$), followed by beautiful ($I = 0.526$), safe ($I = 0.509$), depressing ($I = 0.482$), and wealthy ($I = 0.472$). These results indicate that neighboring CBGs tend to have similar values of the model-implied perception-conditioned relative advantage rather than being randomly distributed across space.

Table S10: Global Moran's I for perception-conditioned relative advantage surfaces.

**Perception dimension**	**Moran's I**	**Permutation p-value**	**z-score**
Beautiful	0.5259	< 0.001	68.644
Livable	0.6649	< 0.001	86.775
Safe	0.5088	< 0.001	67.341
Wealthy	0.4720	< 0.001	61.033
Boring	0.7099	< 0.001	92.875
Depressing	0.4824	< 0.001	62.589

Permutation significance was calculated using 9,999 random permutations under a row-standardized Queen-contiguity spatial-weight matrix. Four spatial islands were excluded, resulting in $N = 5{,}746$ CBGs.

3.3.2 Local spatial association

While Global Moran's I evaluates overall spatial autocorrelation, it does not indicate where significant spatial concentrations occur. We therefore estimated Local Moran's I, or Local Indicators of Spatial Association (LISA), for each CBG:

$$I_i = z_i \sum_j w_{ij} z_j, \tag{14}$$

where z_i denotes the standardized value for CBG i, and $\sum_j w_{ij} z_j$ represents the spatial lag among neighboring CBGs.

Local significance was assessed using 9,999 permutations. To account for the large number of simultaneous local tests, permutation p-values were adjusted using the Benjamini–Hochberg false discovery rate (FDR) procedure with a significance threshold of $q < 0.05$. A fixed random seed of 42 was used for reproducibility.

Significant CBGs were classified into four conventional LISA categories. High–High (HH) areas have relatively high values surrounded by high-valued neighbors, whereas Low–Low (LL) areas have relatively low values surrounded by similarly low-valued neighbors. High–Low (HL) and Low–High (LH) areas represent spatial outliers, where the value of a CBG differs from that of its surrounding neighbors.

Across all six perception dimensions, significant HH and LL clusters were substantially more common than HL or LH spatial outliers (**Table S11**). This pattern is consistent

with the significant positive Global Moran's I values and indicates that the spatial structure is primarily characterized by geographically contiguous concentrations of similar values rather than isolated local anomalies.

Table S11: Local Moran's I cluster classifications.

**Perception**	**High–High**	**Low–Low**	**Low–High**	**High–Low**	**Not sig.**
Beautiful	457	515	51	38	4,685
Livable	847	940	68	32	3,859
Safe	511	432	43	42	4,718
Wealthy	492	403	47	52	4,752
Boring	962	1,060	31	41	3,652
Depressing	411	471	51	46	4,767

Local significance was evaluated using 9,999 permutations and Benjamini–Hochberg false-discovery-rate correction at $q < 0.05$. Counts refer to the $N = 5{,}746$ CBGs included in the spatial analysis.

The strongest local clustering was observed for boring and livable perceptions. For boring, 962 CBGs were classified as significant High–High clusters and 1,060 as Low–Low clusters, whereas only 31 and 41 CBGs were classified as Low–High and High–Low spatial outliers, respectively. Livability similarly exhibited 847 High–High and 940 Low–Low CBGs, compared with 68 Low–High and 32 High–Low outliers. These local patterns are consistent with the comparatively high Global Moran's I values obtained for these two dimensions.

Beauty, safety, wealth, and depressingness also exhibited statistically significant local spatial associations, although a larger proportion of CBGs was classified as non-significant after FDR correction. For beauty, 972 CBGs formed significant High–High or Low–Low clusters; the corresponding totals were 943 for safety, 895 for wealth, and 882 for depressingness. Across all dimensions, therefore, significant spatial association was dominated by same-sign clustering rather than spatial outliers.

3.3.3 Interpretation in relation to Figure 3c

These spatial-autocorrelation results provide formal statistical support for the non-random geographic structure visible in Figure 3c. The Global Moran's I statistics demonstrate significant positive spatial dependence across all six perception-conditioned surfaces, while the LISA analysis identifies localized concentrations of similar values and shows that High–High and Low–Low clusters dominate over spatial outliers.

Importantly, these results should be interpreted in relation to the construction of Figure 3c. The mapped quantity represents the relative sensitivity of protected versus painted bicycle-lane effects to each perception dimension, rather than a direct observation of treatment effects at the CBG level. Because the mapped relative advantage is obtained by applying the estimated moderation-coefficient difference to the spatial distribution of standardized perception scores, the observed spatial clustering reflects the geographic structure of the perception-conditioned model predictions. Accordingly, the Moran and LISA analyses establish that these model-implied spatial patterns are significantly structured, but they do not provide an independent causal test of geographically clustered cycling responses.

Taken together, the global and local spatial analyses complement Figure 3c by quantifying the extent and location of spatial dependence in the model-implied perception-conditioned relative advantages of protected and painted bicycle infrastructure. In particular, the comparatively strong clustering observed for boringness and livability indicates that these perception dimensions form geographically coherent spatial contexts across New York City, within which the relative predicted performance of bicycle-lane types also varies systematically.

3.4 Predicted Treatment Effects Across the Perception Distribution

To translate the estimated moderation coefficients into substantively interpretable outcomes, we compute predicted treatment effects across the observed distribution of each perception dimension. This approach allows us to evaluate how the effectiveness of bicycle infrastructure varies continuously across different perceived neighborhood contexts.

For each lane type l and perception dimension k, the predicted treatment effect is given by:

$$\hat{\tau}^{(l)}(z_k) = \hat{\alpha}^{(l)} + \hat{\beta}_k^{(l)} z_k \tag{15}$$

where z_k denotes the standardized perception score. By construction, $z_k = 0$ represents the citywide average perception level, while positive and negative values correspond to more and less strongly perceived environments, respectively. All other perception variables are held at their mean values to isolate the effect of a single perception dimension. This specification captures the total expected impact of bicycle infrastructure, combining both the baseline effect ($\hat{\alpha}^{(l)}$) and the perception-dependent adjustment ($\hat{\beta}_k^{(l)} z_k$). By tracing predicted treatment effects across the full range of perception scores, we identify how ridership gains vary across different urban contexts (see **Figure 3**). For clarity, perception values are grouped into negative ($z_k < -1$), neutral ($-1 \leq z_k \leq 1$), and positive ($z_k > 1$) categories, summarizing how infrastructure performance differs across less favorable to highly favorable environments. Comparing painted and protected lanes highlights where lower-cost interventions may suffice and where physically separated infrastructure yields larger gains. To further isolate the role of perception, we also examine the incremental change in the treatment effect associated with variation in perception dimension k, independent of the baseline effect $\hat{\alpha}^{(l)}$. These marginal effects are presented in Supplementary **Figure S8**, highlighting how perception alone contributes to treatment heterogeneity across lane types.

3.5 Safety–neighborhood soundness classification of street-level scenarios

To characterize the streetscape contexts represented by the four street-level scenarios in Figure 4, we classified candidate street segments using a two-by-two typology based on (1) general visually perceived safety and (2) overall perceived neighborhood soundness.

The neighborhood-soundness dimension was adapted from the Neighborhood Soundness Index (NSI) proposed by Wang et al. (2025)[8]. In that framework, the six human-perception dimensions are combined into a composite measure of perceived neighborhood quality, with beautiful, livable, wealthy, and safe treated as positively oriented dimensions and boring and depressing reverse-coded before aggregation. The original NSI assigns equal weight to each component perception.

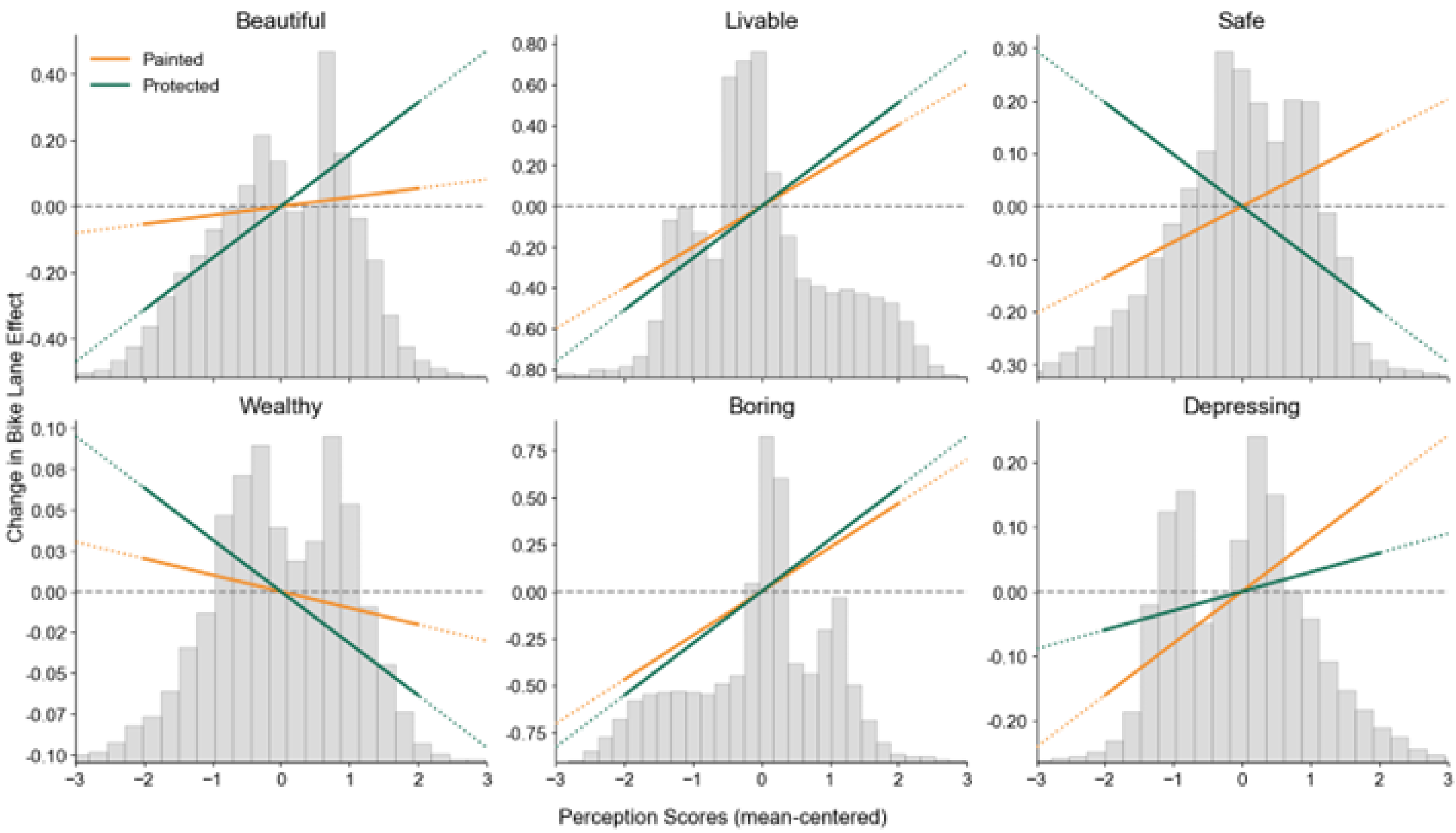

Figure S8: Incremental Effects of Perceived Streetscape Characteristics on Bicycle Infrastructure Impacts

Because general visually perceived safety is used separately as the first dimension of our two-by-two classification, we constructed a safety-excluded NSI for the second dimension to avoid incorporating the same safety measure on both axes. Let $q_{m,s}$ denote the normalized perception score for street segment s and perception dimension m. The modified neighborhood soundness index was calculated as

$$NSI_s^{(-S)} = \frac{q_{\text{beautiful},s} + q_{\text{livable},s} + q_{\text{wealthy},s} + (1 - q_{\text{boring},s}) + (1 - q_{\text{depressing},s})}{5}, \tag{16}$$

where the perception scores were normalized to a common $[0, 1]$ scale before aggregation. Higher values therefore indicate street environments that are, on average, perceived as more beautiful, livable, and wealthy, and less boring and depressing.

For comparability across candidate segments, both visually perceived safety and the modified NSI were subsequently standardized relative to their NYC-wide distributions:

$$z_{\text{safe},s} = \frac{q_{\text{safe},s} - \mu_{\text{safe}}}{\sigma_{\text{safe}}}, \tag{17}$$

and

$$z_{\text{NSI},s} = \frac{NSI_s^{(-S)} - \mu_{\text{NSI}}}{\sigma_{\text{NSI}}}. \tag{18}$$

A value greater than zero therefore indicates an above-average perception relative to the NYC-wide distribution, whereas a value at or below zero indicates an at- or below-average perception.

Combining the two dimensions yields four street-context categories:

1. higher perceived safety–higher neighborhood soundness;

2. higher perceived safety–lower neighborhood soundness;
3. lower perceived safety–higher neighborhood soundness; and
4. lower perceived safety–lower neighborhood soundness.

The four corridors shown in Figure 4 were selected to represent these contrasting combinations. Riverside Drive represents a higher-safety–higher-soundness context, whereas Vanderbilt Avenue represents a higher-safety–lower-soundness context. Madison Avenue represents a lower-safety–higher-soundness context, while West 125th Street represents a lower-safety–lower-soundness context (**Table 12**).

Table S12: Safety–neighborhood soundness classification of the four street-level scenarios shown in Figure 4.

**Corridor**	z_{safe}	z_{NSI}	**Context**	**Painted (%)**	**Protected (%)**
Vanderbilt Ave.	+0.53	-0.28	Higher safety–Lower NSI	11.4	21.2
Riverside Dr.	+0.71	+0.62	Higher safety–Higher NSI	10.3	28.7
Madison Ave.	-0.34	+0.44	Lower safety–Higher NSI	1.9	33.8
West 125th St.	-0.66	-0.49	Lower safety–Lower NSI	2.8	34.9

The scenario results reveal a consistent contrast across the safety dimension. Vanderbilt Avenue and Riverside Drive, both characterized by above-average visually perceived safety, show comparatively more modest differences between the predicted effects of painted and protected bicycle-lane scenarios. This indicates that painted lanes can still generate meaningful ridership gains in street environments where the baseline visual context is already relatively supportive. In contrast, Madison Avenue and West 125th Street, both characterized by below-average visually perceived safety, exhibit substantially larger differences between the two lane scenarios, with protected infrastructure generating considerably larger predicted gains. The comparison therefore suggests that the relative benefit of physical separation is greatest in street contexts where the baseline environment is perceived as less safe, while the NSI dimension further distinguishes whether these streets otherwise exhibit higher or lower overall perceived neighborhood soundness.

References

[1] Joseph Broach, Jennifer Dill, and John Gliebe. Where do cyclists ride? a route choice model developed with revealed preference gps data. *Transportation Research Part A: Policy and Practice*, 46(10):1730–1740, 2012.

[2] K. Ito, Y. Zhu, M. Abdelrahman, X. Liang, Z. Fan, Y. Hou, others, and F. Biljecki. Zensvi: An open-source software for the integrated acquisition, processing and analysis of street view imagery towards scalable urban science. *Computers, Environment and Urban Systems*, 119:102283, 2025.

[3] Mapillary. Mapillary api documentation. `https://www.mapillary.com/developer/api-documentation`, 2025. Accessed 2025.

[4] Nikhil Naik, Julie Philipoom, Ramesh Raskar, and Cesar Hidalgo. Streetscore—predicting the perceived safety of one million streetscapes. In *Proceedings of the IEEE Conference on Computer Vision and Pattern Recognition Workshops*, pages 779–785, 2014.

[5] Roberto Nacxit Ramirez Juarez, Anna Beatriz Grigolon, and Ana Mafalda Madureira. Cyclists' perception of streetscape and its influence on route choice: A pilot study with a mixed-methods approach. *Transportation Research Part F: Traffic Psychology and Behaviour*, 99:374–388, 2023.

[6] U.S. Census Bureau. American community survey (acs), 2022. Available at: `https://www.census.gov/programs-surveys/acs`.

[7] U.S. Environmental Protection Agency. Smart location database, 2022. Available at: `https://www.epa.gov/smartgrowth/smart-location-mapping`.

[8] S. Wang, J. Yoo, W. Cai, F. Yang, X. Huang, Q. C. Sun, and S. Lyu. Reducing the social inequity of neighborhood visual environment in los angeles through computer vision and multi-model machine learning. *Sustainable Cities and Society*, 119:106062, 2025.

[9] Meghan Winters, Gavin Davidson, Diana Kao, and Kay Teschke. Motivators and deterrents of bicycling: comparing influences on decisions to ride. *Transportation*, 38(1):153–168, 2011.

[10] Lurong Xu, Shuli Luo, Alexa Delbosc, Steve O'Hern, and Zhuo Chen. Non-linear effects of built environment on cyclists' perceived safety and comfort using online pairwise voting. *Transportation Research Part A: Policy and Practice*, 205:104850, 2026.

[11] C. Yang and H. Guo. A method of image semantic segmentation based on pspnet. *Mathematical Problems in Engineering*, 2022(1):8958154, 2022.
